# Stability of a discrete HTLV-1/SARS-CoV-2 dual infection model

**DOI:** 10.1016/j.heliyon.2024.e28178

**Published:** 2024-03-18

**Authors:** M.A. Alshaikh, A.K. Aljahdali

**Affiliations:** a Department of Mathematics, College of Science, Taif University, P.O. Box 11099, Taif 21944, Saudi Arabia; b Department of Mathematics, Faculty of Science, King Abdulaziz University, P. O. Box 80203, Jeddah 21589, Saudi Arabia

**Keywords:** Non-standard discretization, HTLV-1/SARS-CoV-2 dual infection, Lyapunov method

## Abstract

Dual infection with a virus that targets the immune system, such as HTLV-1 (human T-cell lymphotropic virus class 1), combined with another virus that affects the respiratory system, such as SARS-CoV-2 (severe acute respiratory syndrome coronavirus 2), can cause serious disease and even death. Given the significance of better comprehending the dual viral infections' dynamics, researchers have been drawn to mathematical analyses of such models. This work investigates the stability of a discrete HTLV-1/SARS-CoV-2 dual infection model. Our approach involves formulating the discrete model through the discretization of the continuous-time one using NSFD (nonstandard finite difference) method. We demonstrate that the NSFD method preserves essential properties of the solutions, such as positivity and boundedness. Additionally, we determine the fixed points and establish the conditions under which they exist. Furthermore, we analyze the global stability of these fixed points utilizing the Lyapunov technique. To illustrate our analytical findings, we do numerical simulations.

## 1 Introduction

HTLV-1 (Human T-cell lymphotropic virus class 1) is one of the deadliest viruses known to affect the human body, potentially resulting in fatal outcomes. It has the ability to infect a variety of cell types, such as dendritic cells, macrophages, monocytes, and CTLs (cytotoxic T lymphocytes), but its main target is CD4^+^T cells [1]. Two disorders linked to HTLV-1 infection include ATL (adult T-cell leukemia) and HAM/TSP (HTLV-1-associated myelopathy/tropical spastic paraparesis) [2]. COVID-19 (Coronavirus Disease 2019) has claimed millions of lives and is caused by SARS-CoV-2, which specifically attacks the ECs (epithelial cells) of the respiratory tract [3].

Examining the mathematical modeling of virus infection dynamics in the host can be immensely beneficial in understanding the dynamic interactions among the virus, immune cells, and target cells. The efficacy of drugs, either alone or in combination, is another topic this study contributes to our understanding of HTLV-1 mono-infection model with latently HTLV-infected cells was developed in [4] and [5]. Several biological processes were integrated into HTLV mono-infection models, including (i) CTL immunity [6], [7], [8], [9], (ii) mitotic transmission [10], [11], [12], and (iii) time delays [7], [13], [14]. Assuming a limited number of target cells, a model for SARS-CoV-2 mono-infection was created in [15]. Additionally, the generation and mortality of target cells were modeled in [16]. Models presented in [15], [16] were extended by including the impact of immunity response [17], [18], [19], [20], treatment [21], [22], [23], [24], time delay [25] and reaction-diffusion [26].

There are signs that the prevalence of HTLV-1 infection is rising in correlation with other blood-borne illnesses, (hepatitis B (and C) viruses, HIV-1 (human immunodeficiency virus class-1)) and sexually transmitted diseases (HPV (human papillomavirus), syphilis, HSV (Herpes simplex virus), chlamydia) [27]. Recent works (see [28], [29], [30], [31]) reported cases infected with both SARS-CoV-2 and HTLV-1. In [32], the following HTLV-1/SARS-CoV-2 co-dynamics model was formulated:

Healthy ECs:

(1) $$\overset{˙}{X}=\underset{\text{production of healthy ECs}}{\underset{︸}{\xi }}-\underset{\text{death}}{\underset{︸}{\phi_{X}X}}-\underset{\text{SARS-CoV-2/ECs incidence}}{\underset{︸}{\varrho VX}}.$$

Latently SARS-CoV-2-infected ECs:

(2) $$\overset{˙}{N}=\underset{\text{SARS-CoV-2/ECs incidence}}{\underset{︸}{\varrho VX}}-\underset{\text{latent-to-active transmission}}{\underset{︸}{\rho N}}-\underset{\text{death}}{\underset{︸}{\phi_{N}N}}.$$

Actively SARS-CoV-2-infected ECs:

(3) $$\overset{˙}{Y}=\underset{\text{latent-to-active transmission}}{\underset{︸}{\rho N}}-\underset{\text{death}}{\underset{︸}{\phi_{Y}Y}}-\underset{\text{killing of actively SARS-CoV-2-infected ECs}}{\underset{︸}{\kappa YU}}.$$

Free SARS-CoV-2 particles:

(4) $$\overset{˙}{V}=\underset{\text{generation of SARS-CoV-2}}{\underset{︸}{\mu Y}}-\underset{\text{death}}{\underset{︸}{\phi_{V}V}}.$$

Healthy CD4^+^T cells:

(5) $$\overset{˙}{U}=\underset{\text{production of healthy CD4}{}^{+}\text{T cells}}{\underset{︸}{\eta }}+\underset{\text{stimulation of healthy CD4}{}^{+}\text{T cells}}{\underset{︸}{\varkappa YU}}-\underset{\text{death}}{\underset{︸}{\phi_{U}U}}-\underset{\text{infection of CD4}{}^{+}\text{T cells by HTLV-1}}{\underset{︸}{\lambda AU}}.$$

Latently HTLV-infected CD4^+^T cells:

(6) $$\overset{˙}{L}=\underset{\text{infection of CD4}{}^{+}\text{T cells by HTLV-1}}{\underset{︸}{\lambda AU}}+\underset{\text{miotic transmission}}{\underset{︸}{\theta \omega^{⁎}A}}-\underset{\text{latent-to-active transmission}}{\underset{︸}{\nu L}}-\underset{\text{death}}{\underset{︸}{\phi_{L}L}}.$$

Actively HTLV-infected CD4^+^T cells:

(7) $$\overset{˙}{A}=\underset{\text{latent-to-active transmission}}{\underset{︸}{\nu L}}+\underset{\text{proliferation of actively HTLV-infected CD4}{}^{+}\text{T cells}}{\underset{︸}{(1-\theta )\omega^{⁎}A}}-\underset{\text{death}}{\underset{︸}{\phi_{A}^{⁎}A}}.$$

Parameters *ξ*, $\phi_{X}$, *ϱ*, *ρ*, $\phi_{N}$, $\phi_{Y}$, *κ*, *μ*, $\phi_{V}$, *η*, *ϰ*, $\phi_{U}$, *λ*, *θ*, $\omega^{⁎}$, *ν*, $\phi_{L}$ and $\phi_{A}^{⁎}$ are positive and θ∈(0,1). It was assumed in [11] that, $\omega^{⁎}<\min\left\{\phi_{U},\phi_{L},\phi_{A}^{⁎}\right\}$. Since 0<θ<1 and $\omega^{⁎}<\phi_{A}^{⁎}$, then $(1-\theta )\omega^{⁎}<\phi_{A}^{⁎}$. Define $\phi_{A}=$ $\phi_{A}^{⁎}-(1-\theta )\omega^{⁎}>0$ and $\omega =\theta \omega^{⁎}$. Therefore, model (1)-(7) becomes

(8) $$\overset{˙}{X}=\xi -\phi_{X}X-\varrho VX,$$

(9) $$\overset{˙}{N}=\varrho VX-(\rho +\phi_{N})N,$$

(10) $$\overset{˙}{Y}=\rho N-\phi_{Y}Y-\kappa YU,$$

(11) $$\overset{˙}{V}=\mu Y-\phi_{V}V,$$

(12) $$\overset{˙}{U}=\eta +\varkappa YU-\phi_{U}U-\lambda AU,$$

(13) $$\overset{˙}{L}=\lambda AU+\omega A-(\nu +\phi_{L})L,$$

(14) $$\overset{˙}{A}=\nu L-\phi_{A}A.$$

The models reported in the literature for HTLV-1 mono-infection, SARS-CoV-2 mono-infection, and HTLV-1/SARS-CoV-2 dual infection are all observed to be represented by nonlinear continuous-time ODEs. Nonetheless, for the following reasons, discrete-time models are more suited to explain viral infection [33]:

• The exact analytical solutions of most nonlinear continuous-time systems for viral infection are unknown, therefore discretization is inevitable.
• Discrete-time system research became necessary due to the use of digital computers in simulations to forecast the course of viruses, the immune system's reaction, and the efficacy of treatments.
• Real data from virally infected people is typically gathered and analyzed by scientists at discrete time instants.

Thus, only approximate discrete-time solutions can be obtained for most nonlinear viral infection systems. Consequently, it makes sense to describe the dynamics of virus infection using the discrete-time model. In fact, the discretization technique has to preserve the key characteristics of their continuous-time equivalents, namely stability and equilibria. Numerical instability and bias arise when discretizing nonlinear continuous-time models using classical techniques like Runge-Kutta, Euler, and others when using high step sizes [34]. Furthermore, these techniques could result in a forecast of dynamic behavior that is incorrect, such as a convergence to the incorrect fixed point or periodic cycle [33]. Preserving positivity is a crucial component of virological and epidemiological models [35]. However, retaining non-negativity for arbitrarily large time step sizes is a drawback of many classical time discretization systems [35], [36]. It has been demonstrated by Gyurkovics and Elaiw [37] that in the case of nonlinear control systems, the controller derived from the approximation-based design may stabilize the approximate closed-loop system model while destabilizing the original model. Therefore, one has to use a discretization method which preserves the continuous-time model's key qualitative attributes. Mickens [38] presented NSFD (non-standard finite difference) scheme for solving differential equations. NSFD method was successfully used in describing and analyzing several viral infection models in both epidemiology [39], [40], [41], [42], [43], [44], [45] and virology [46], [47], [48], [49], [50], [51], [52], [53], [54], [55], [56], [57], [58], [59]. NSFD has been applied to models of the dynamics of within-host viruses, such as: (i) ordinary differential equations, [46], [47], (ii) delay differential equations [48], (iii) partial differential equations [34], [49], [50], [51], [52] and (iv) delay partial differential equations [53], [54]. It has been shown that, regardless of the step-size used, NSFD has the benefit of maintaining the key qualitative characteristics of these models, such as positivity/boundedness pf solutions, and stability of equilibria.

We note that in the above mentioned works, NSFD was used for discretizing continuous-time models for viral mono-infection. In very recent works [60] and [61], NSFD was used for solving HIV-1/HTLV-I dual infection models. Discretization of the continuous-time models of simultaneous infection with SARS-CoV-2 and HTLV-1 has not, however, been examined previously. Our current effort aims to discretize an HTLV-1/SARS-CoV-2 dual infection model using the NSFD approach. We demonstrate the positivity and ultimate boundedness of the discrete HTLV-1/SARS-CoV-2 dual infection model solutions. We identify four model fixed points and determine the conditions in which they exist based on four threshold parameters. ($TP_{i}$, i=1,2,3,4). For discrete-time systems, we employ the Lyapunov approach to demonstrate the global stability of every fixed point. To put the acquired theoretical conclusions into practice, we do numerical simulations.

## 2 The discrete HTLV-1/SARS-CoV-2 dual infection model

In this section, we discretize model (8)-(14) by using NSFD approach. Let $\left(X_{n},N_{n},Y_{n},V_{n},U_{n},L_{n},A_{n}\right)$ be an approximation of solution of system (8)-(14) at the time instants $t_{n}=nd$, n={0,1,2,...} and $d=t_{n+1}-t_{n}>0$ is the time step size. A finite difference scheme is considered nonstandard, according to Mickens, if at least one of the following criteria is met [38], [62], [63], [64], [65]:

• The approximation is nonlocal.

• The derivative is discretized using a denominator function, which is not a conventional method $0<B(d)=d+O(d^{2})$. Applying NSFD scheme on model (8)-(14) we get:

(15) $$\frac{X_{n+1}-X_{n}}{B(d)}=\xi -\phi_{X}X_{n+1}-\varrho V_{n}X_{n+1},$$

(16) $$\frac{N_{n+1}-N_{n}}{B(d)}=\varrho V_{n}X_{n+1}-(\rho +\phi_{N})N_{n+1},$$

(17) $$\frac{Y_{n+1}-Y_{n}}{B(d)}=\rho N_{n+1}-\phi_{Y}Y_{n+1}-\kappa Y_{n+1}U_{n+1},$$

(18) $$\frac{V_{n+1}-V_{n}}{B(d)}=\mu Y_{n+1}-\phi_{V}V_{n+1},$$

(19) $$\frac{U_{n+1}-U_{n}}{B(d)}=\eta +\varkappa Y_{n+1}U_{n+1}-\phi_{U}U_{n+1}-\lambda A_{n}U_{n+1},$$

(20) $$\frac{L_{n+1}-L_{n}}{B(d)}=\lambda A_{n}U_{n+1}+\omega A_{n}-(\nu +\phi_{L})L_{n+1},$$

(21) $$\frac{A_{n+1}-A_{n}}{B(d)}=\nu L_{n+1}-\phi_{A}A_{n+1}.$$

The denominator function B(d) is defined as [62], [64]:

$$B(d)=\frac{1-e^{-\phi_{X}d}}{\phi_{X}}.$$

The system's initial conditions are

(22) $$(X_{0},N_{0},Y_{0},V_{0},U_{0},L_{0},A_{0})\in R_{+}^{7}=\{(X,N,Y,V,U,L,A)|X>0,N>0,Y>0,V>0,U>0,L>0,A>0\}.$$

## 3 Preliminaries

We verify the biological acceptability of our model by determining a restricted domain for the concentrations of its compartments. In particular, there shouldn't be any negative or infinite concentrations.

Let $\sigma =\min\left\{\phi_{X},\phi_{N},\frac{\phi_{Y}}{2},\phi_{V},\phi_{U},\phi_{L},\frac{\phi_{A}^{⁎}-\omega^{⁎}}{1+\theta \omega^{⁎}}\right\}$ and $W_{1}=\frac{\xi }{\sigma }+\frac{\kappa \eta }{\sigma \varkappa },W_{2}=\frac{2\mu }{\phi_{Y}}W_{1},W_{3}=\frac{\varkappa }{\kappa }W_{1}$ and define the region $\Gamma =\{(X,N,Y,V,U,L,A):0<X,N,Y<W_{1},0<V<W_{2},0<U,L,A<W_{3}\}$.

Lemma 1 *Any solution of the discrete model* (15) *-* (21) *under initial conditions* (22) *is positive and ultimately bounded.*

Proof Eqs. (15)-(21) yield

(23) $$X_{n+1}=\frac{B(d)\xi +X_{n}}{1+B(d)(\phi_{X}+\varrho V_{n})},$$

(24) $$N_{n+1}=\frac{B(d)\varrho V_{n}X_{n+1}+N_{n}}{1+B(d)(\rho +\phi_{N})},$$

(25) $$Y_{n+1}=\frac{B(d)\rho N_{n+1}+Y_{n}}{1+B(d)(\phi_{Y}+\kappa U_{n+1})},$$

(26) $$V_{n+1}=\frac{B(d)\mu Y_{n+1}+V_{n}}{1+B(d)\phi_{V}},$$

(27) $$U_{n+1}=\frac{B(d)\eta +U_{n}}{1+B(d)(\phi_{U}-\varkappa Y_{n+1}+\lambda A_{n})},$$

(28) $$L_{n+1}=\frac{B(d)\lambda A_{n}U_{n+1}+B(d)\omega A_{n}+L_{n}}{1+B(d)(\nu +\phi_{L})},$$

(29) $$A_{n+1}=\frac{B(d)\nu L_{n+1}+A_{n}}{1+B(d)\phi_{A}}.$$

We show that ($X_{1},N_{1},Y_{1},V_{1},U_{1},L_{1},A_{1})$ exists, positive and unique. If n=0, then from Eqs. (23) and (24) and the initial state (22), we obtain $X_{1}>0$ and $N_{1}>0$. From Eqs. (25) and (27), we obtain

$$U_{1}=\frac{\left[B(d)\eta +U_{0}\right]\left[1+B(d)(\phi_{Y}+\kappa U_{1})\right]}{\left[1+B(d)(\phi_{U}+\lambda A_{0})\right]\left[1+B(d)(\phi_{Y}+\kappa U_{1})\right]-\varkappa B(d)\left[B(d)\rho N_{1}+Y_{0}\right]}.$$

Then

(30) $$A_{1}U_{1}^{2}+B_{1}U_{1}+C_{1}=0,$$

where

$$A_{1}=\kappa B(d)(1+B(d)\phi_{U}+B(d)\lambda A_{0}),B_{1}=\left[1+B(d)(\phi_{U}+\lambda A_{0})\right]\left[1+B(d)\phi_{Y}\right]-\varkappa B(d)\left[B(d)\rho N_{1}+Y_{0}\right]-\kappa B(d)\left[B(d)\eta +U_{0}\right],C_{1}=-\left[(1+B(d)\phi_{Y})(B(d)\eta +U_{0})\right].$$

Since $A_{1}>0$ and $C_{1}<0$, then $B_{1}^{2}-4A_{1}C_{1}>0$. This implies that Eq. (30) has a unique root $U_{1}>0$. From Eqs. (25), (26), (28) and (29), we obtain

$$Y_{1}=\frac{B(d)\rho N_{1}+Y_{0}}{1+B(d)(\phi_{Y}+\kappa U_{1})}>0.V_{1}=\frac{B(d)\mu Y_{1}+V_{0}}{1+B(d)\phi_{V}}>0,L_{1}=\frac{B(d)\lambda A_{0}U_{1}+B(d)\omega A_{0}+L_{0}}{1+B(d)(\nu +\phi_{L})}>0,A_{1}=\frac{B(d)\nu L_{1}+A_{0}}{1+B(d)\phi_{A}}>0.$$

Therefore, the solution $\left(X_{1},N_{1},Y_{1},V_{1},U_{1},L_{1},A_{1}\right)$ exists, positive and unique. We can demonstrate that $\left(X_{2},N_{2},Y_{2},V_{2},U_{2},L_{2},A_{2}\right)$ exists uniquely and is positive by repeating the previous procedure for n=1.

Mathematical induction provides that for all n≥0, $\left(X_{n},N_{n},Y_{n},V_{n},U_{n},L_{n},A_{n}\right)$ exists, positive and unique.

Define a sequence $M_{n}$:

$$M_{n}=X_{n}+N_{n}+Y_{n}+\frac{\phi_{Y}}{2\mu }V_{n}+\frac{\kappa }{\varkappa }(U_{n}+L_{n}+(1+\theta \omega^{⁎})A_{n}).$$

Then

$$M_{n+1}-M_{n}=(X_{n+1}-X_{n})+(N_{n+1}-N_{n})+(Y_{n+1}-Y_{n})+\frac{\phi_{Y}}{2\mu }(V_{n+1}-V_{n})+\frac{\kappa }{\varkappa }\left[(U_{n+1}-U_{n}\right)+(L_{n+1}-L_{n})+(1+\theta \omega^{⁎})(A_{n+1}-A_{n})]=B(d)[\xi -\phi_{X}X_{n+1}-\phi_{N}N_{n+1}-\frac{\phi_{Y}}{2}Y_{n+1}-\frac{\phi_{Y}}{2\mu }\phi_{V}V_{n+1}+\frac{\kappa }{\varkappa }\eta -\frac{\kappa \phi_{U}}{\varkappa }U_{n+1}-\frac{\kappa \phi_{L}}{\varkappa }L_{n+1}-\frac{\kappa }{\varkappa }(\phi_{A}^{⁎}-\omega^{⁎})A_{n+1}]\leq B(d)\left(\xi +\frac{\kappa }{\varkappa }\eta \right)-B(d)\sigma [X_{n+1}+N_{n+1}+Y_{n+1}+\frac{\phi_{Y}}{2\mu }V_{n+1}+\frac{\kappa }{\varkappa }(U_{n+1}+L_{n+1}+(1+\theta \omega^{⁎})A_{n+1}\;)]=B(d)\left(\xi +\frac{\kappa }{\varkappa }\eta \right)-B(d)\sigma M_{n+1}.$$

Hence

$$M_{n+1}\leq \frac{M_{n}}{1+B(d)\sigma }+\frac{B(d)\left(\frac{\xi \varkappa +\kappa \eta }{\varkappa }\right)}{1+B(d)\sigma }.$$

Lemma 2.2 in [66] gives

$$M_{n}\leq \frac{\xi \varkappa +\kappa \eta }{\varkappa \sigma }\left(1-\frac{1}{{\left(1+B(d)\sigma \right)}^{n}}\right)+\frac{M_{0}}{{\left(1+B(d)\sigma \right)}^{n}}.$$

This gives, $\underset{n\to \infty }{\lim\;\sup}\;M_{n}\leq W_{1}$, and thus $\underset{n\to \infty }{\lim\;\sup}(X_{n},N_{n},Y_{n},V_{n},U_{n},L_{n},A_{n})\leq (W_{1},W_{1},W_{1},W_{2},W_{3},W_{3},W_{3})$. Therefore, $\left(X_{n},N_{n},Y_{n},V_{n},U_{n},L_{n},A_{n}\right)$ converges to Γ as n→∞.

## 4 Fixed points

The fixed points of (15)-(21) satisfy the following:

$$0=\xi -\phi_{X}X-\varrho VX,0=\varrho VX-(\rho +\phi_{N})N,0=\rho N-\phi_{Y}Y-\kappa YU,0=\mu Y-\phi_{V}V,0=\eta +\varkappa YU-\phi_{U}U-\lambda AU,0=\lambda AU+\omega A-(\nu +\phi_{L})L,0=\nu L-\phi_{A}A$$

We find that the system admits four fixed points.

1) Infection-free fixed point $FP_{0}=(X^{0},0,0,0,U^{0},0,0)$, where $X^{0}=\frac{\xi }{\phi_{X}}$ and $U^{0}=\frac{\eta }{\phi_{U}}$, which always exists.

2) HTLV-1 mono-infection fixed point $FP_{1}=(\overset{ˆ}{X},0,0,0,\overset{ˆ}{U},\overset{ˆ}{L},\overset{ˆ}{A})$, where

$$\overset{ˆ}{X}\;=X^{0}=\frac{\xi }{\phi_{X}},\;\;\;\overset{ˆ}{U}=\frac{\phi_{L}\phi_{A}+\nu (\phi_{A}-\omega )}{\nu \lambda }=\frac{U^{0}}{TP_{1}},\overset{ˆ}{L}=\frac{\phi_{U}\phi_{A}}{\nu \lambda }\left[\frac{\nu \eta \lambda }{\phi_{U}(\phi_{L}\phi_{A}+\nu (\phi_{A}-\omega ))}-1\right]=\frac{\phi_{U}\phi_{A}}{\nu \lambda }(TP_{1}-1),\overset{ˆ}{A}=\frac{\phi_{U}}{\lambda }\left[\frac{\nu \eta \lambda }{\phi_{U}(\phi_{L}\phi_{A}+\nu (\phi_{A}-\omega ))}-1\right]=\frac{\phi_{U}}{\lambda }(TP_{1}-1),$$

where

$$TP_{1}=\frac{\nu \eta \lambda }{\phi_{U}(\phi_{L}\phi_{A}+\nu (\phi_{A}-\omega ))},$$

is the threshold parameter $TP_{1}$ represents the basic reproduction number of HTLV-1 mono-infection. It establishes the presence of HTLV-1 infection. Since $\phi_{A}-\omega >0$, then $TP_{1}$ is positive. We have $\overset{ˆ}{L}>0$ and $\overset{ˆ}{A}>0$ if $TP_{1}>1$. Therefore, $FP_{1}$ exists when $TP_{1}>1$.

3) SARS-CoV-2 mono-infection fixed point $FP_{2}=(\overset{˜}{X},\overset{˜}{N},\overset{˜}{Y},\overset{˜}{V},\overset{˜}{U},0,0)$ where

(31) $$\overset{˜}{Y}=\frac{\phi_{V}}{\mu }\overset{˜}{V},\;\overset{˜}{U}=\frac{\mu \eta }{\mu \phi_{U}-\varkappa \phi_{V}\overset{˜}{V}},\;\overset{˜}{X}=\frac{\left(\rho +\phi_{N})(\overset{˜}{Y}\phi_{Y}+\overset{˜}{U}\overset{˜}{Y}\kappa \right)}{\rho \varrho \overset{˜}{V}},\;\overset{˜}{N}=\frac{\overset{˜}{Y}\phi_{Y}+\overset{˜}{U}\overset{˜}{Y}\kappa }{\rho },$$

and $\overset{ˆ}{V}$ satisfies the following equation:

(32) $$\frac{T_{1}{\overset{˜}{V}}^{2}+T_{2}\overset{˜}{V}+T_{3}}{\mu \rho \varrho (\mu \phi_{U}-\varkappa \phi_{V}\overset{˜}{V})}=0,$$

where

$$T_{1}=\varrho \phi_{Y}\phi_{V}^{2}\varkappa (\rho +\phi_{N}),T_{2}=\phi_{X}\phi_{Y}\phi_{V}^{2}\varkappa (\rho +\phi_{N})-\mu \varrho \phi_{Y}\phi_{V}\phi_{U}(\rho +\phi_{N})-\mu \varrho \eta \kappa \phi_{V}(\rho +\phi_{N})-\xi \mu \varrho \rho \varkappa \phi_{V},T_{3}=\xi \mu^{2}\varrho \rho \phi_{U}-\mu \phi_{X}\phi_{Y}\phi_{V}\phi_{U}(\rho +\phi_{N})-\mu \eta \kappa \phi_{X}\phi_{V}(\rho +\phi_{N}).$$

We show that Eq. (32) has a positive root. We define

$$S(V)=\frac{T_{1}V^{2}+T_{2}V+T_{3}}{\mu \rho \varrho (\mu \phi_{U}-\varkappa \phi_{V}V)}.$$

Then

$$S(0)=\frac{\xi \mu^{2}\varrho \rho \phi_{U}-\mu \phi_{X}\phi_{Y}\phi_{V}\phi_{U}(\rho +\phi_{N})-\mu \eta \kappa \phi_{X}\phi_{V}(\rho +\phi_{N})}{\mu^{2}\rho \varrho \phi_{U}}=\frac{\phi_{X}\phi_{V}(\rho +\phi_{N})(\phi_{Y}\phi_{U}+\eta \kappa )}{\mu \rho \varrho \phi_{U}}(TP_{2}-1),$$

where

$$TP_{2}=\frac{\xi \mu \varrho \rho \phi_{U}}{\phi_{X}\phi_{V}(\rho +\phi_{N})(\phi_{Y}\phi_{U}+\eta \kappa )}.$$

This shows that S(0)>0 if $TP_{2}>1$, and

$$\underset{V\to {\left(\frac{\mu \phi_{U}}{\varkappa \phi_{V}}\right)}^{-}}{\lim\;\sup}\;S(V)=-\infty .$$

We have

$$S^{\prime }(V)=-\frac{\phi_{V}(\rho +\phi_{N})}{\mu \rho \varrho {\left(\mu \phi_{U}-V\varkappa \phi_{V}\right)}^{2}}\left[\varrho \phi_{Y}{\left(V\varkappa \phi_{V}-\mu \phi_{U}\right)}^{2}+\mu \eta \varkappa \kappa \phi_{X}\phi_{V}+\mu^{2}\eta \kappa \varrho \phi_{U}\right].$$

Then, $S^{\prime }(V)<0$ for all $V\in \left(0,\frac{\mu \phi_{U}}{\varkappa \phi_{V}}\right)$. Consequently, there's a unique $\overset{˜}{V}\in \left(0,\frac{\mu \phi_{U}}{\varkappa \phi_{V}}\right)$ satisfies $S(\overset{˜}{V})=0$. From Eq. (31), we get that $\overset{˜}{Y}>0$, $\overset{˜}{U}>0$, $\overset{˜}{X}>0$ and $\overset{˜}{N}>0$. As a result, $FP_{2}$ exists when $TP_{2}>1$. The SARS-CoV-2 mono-infection's basic reproduction number is represented by the parameter $TP_{2}$. It confirms that SARS-CoV-2 is a mono-infection.

4) HTLV-1/SARS-CoV-2 dual infection fixed point $FP_{3}=(\overset{¯}{X},\overset{¯}{N},\overset{¯}{Y},\overset{¯}{V},\overset{¯}{U},\overset{¯}{L},\overset{¯}{A})$ where

$$\overset{¯}{X}=\frac{\phi_{V}(\rho +\phi_{N})(\phi_{Y}\nu \lambda +\kappa (\phi_{L}\phi_{A}+\nu (\phi_{A}-\omega )))}{\mu \rho \varrho \nu \lambda },\overset{¯}{N}=\frac{\phi_{X}\phi_{V}(\phi_{Y}\nu \lambda +\kappa (\phi_{L}\phi_{A}+\nu (\phi_{A}-\omega )))}{\mu \rho \varrho \nu \lambda }\left[\frac{\xi \mu \rho \varrho \nu \lambda }{\phi_{X}\phi_{V}(\rho +\phi_{N})(\phi_{Y}\nu \lambda +\kappa (\phi_{L}\phi_{A}+\nu (\phi_{A}-\omega )))}-1\right],\overset{¯}{Y}=\frac{\phi_{X}\phi_{V}}{\mu \varrho }\left[\frac{\xi \mu \rho \varrho \nu \lambda }{\phi_{X}\phi_{V}(\rho +\phi_{N})(\phi_{Y}\nu \lambda +\kappa (\phi_{L}\phi_{A}+\nu (\phi_{A}-\omega )))}-1\right],\overset{¯}{V}=\frac{\phi_{X}}{\varrho }\left[\frac{\xi \mu \rho \varrho \nu \lambda }{\phi_{X}\phi_{V}(\rho +\phi_{N})(\phi_{Y}\nu \lambda +\kappa (\phi_{L}\phi_{A}+\nu (\phi_{A}-\omega )))}-1\right],\overset{¯}{U}=\frac{1}{\nu \lambda }(\phi_{L}\phi_{A}+\nu (\phi_{A}-\omega )),\overset{¯}{L}=\frac{\phi_{A}(\varkappa \phi_{X}\phi_{V}+\mu \varrho \phi_{U})}{\mu \varrho \nu \lambda }\times \left[\frac{\mu \varrho \nu \lambda }{\left(\varkappa \phi_{X}\phi_{V}+\mu \varrho \phi_{U}\right)}\left(\frac{\eta }{\left(\phi_{L}\phi_{A}+\nu (\phi_{A}-\omega )\right)}+\frac{\xi \rho \varkappa }{\left(\rho +\phi_{N})(\phi_{Y}\nu \lambda +\kappa (\phi_{L}\phi_{A}+\nu (\phi_{A}-\omega ))\right)}\right)-1\right],\overset{¯}{A}=\frac{\left(\varkappa \phi_{X}\phi_{V}+\mu \varrho \phi_{U}\right)}{\mu \varrho \lambda }\times \left[\frac{\mu \varrho \nu \lambda }{\left(\varkappa \phi_{X}\phi_{V}+\mu \varrho \phi_{U}\right)}\left(\frac{\eta }{\left(\phi_{L}\phi_{A}+\nu (\phi_{A}-\omega )\right)}+\frac{\xi \rho \varkappa }{\left(\rho +\phi_{N})(\phi_{Y}\nu \lambda +\kappa (\phi_{L}\phi_{A}+\nu (\phi_{A}-\omega ))\right)}\right)-1\right].$$

We have $\phi_{A}-\omega >0$, $\overset{¯}{X}>0$ and $\overset{¯}{U}>0$, while $\overset{¯}{N}>0$, $\overset{¯}{Y}>0$ and $\overset{¯}{V}>0$ if $\frac{\xi \mu \rho \varrho \nu \lambda }{\phi_{X}\phi_{V}(\rho +\phi_{N})(\phi_{Y}\nu \lambda +\kappa (\phi_{L}\phi_{A}+\nu (\phi_{A}-\omega )))}>1$. On the other hand, $\overset{¯}{L}>0$ and $\overset{¯}{A}>0$ when $\frac{\mu \varrho \nu \lambda }{\left(\varkappa \phi_{X}\phi_{V}+\mu \varrho \phi_{U}\right)}\left(\frac{\eta }{\left(\phi_{L}\phi_{A}+\nu (\phi_{A}-\omega )\right)}+\frac{\xi \rho \varkappa }{\left(\rho +\phi_{N})(\phi_{Y}\nu \lambda +\kappa (\phi_{L}\phi_{A}+\nu (\phi_{A}-\omega ))\right)}\right)>1$. Consequently, we are able to rewrite the elements of $FP_{3}$ as:

$$\overset{¯}{X}=\frac{X^{0}}{TP_{4}},\;\;\overset{¯}{N}=\frac{\phi_{X}\phi_{V}(\phi_{Y}\nu \lambda +\kappa (\phi_{L}\phi_{A}+\nu (\phi_{A}-\omega )))}{\mu \rho \varrho \nu \lambda }(TP_{4}-1),\overset{¯}{Y}=\frac{\phi_{X}\phi_{V}}{\mu \varrho }(TP_{4}-1),\;\;\overset{¯}{V}=\frac{\phi_{X}}{\varrho }(TP_{4}-1),\overset{¯}{U}=\frac{1}{\nu \lambda }(\phi_{L}\phi_{A}+\nu (\phi_{A}-\omega )),\;\;\overset{¯}{L}=\frac{\phi_{A}(\varkappa \phi_{X}\phi_{V}+\mu \varrho \phi_{U})}{\mu \varrho \nu \lambda }(TP_{3}-1),\overset{¯}{A}=\frac{\left(\varkappa \phi_{X}\phi_{V}+\mu \varrho \phi_{U}\right)}{\mu \varrho \lambda }(TP_{3}-1),$$

where

$$TP_{3}=\frac{\mu \varrho \nu \lambda }{\left(\varkappa \phi_{X}\phi_{V}+\mu \varrho \phi_{U}\right)}\left(\frac{\eta }{\left(\phi_{L}\phi_{A}+\nu (\phi_{A}-\omega )\right)}+\frac{\xi \rho \varkappa }{\left(\rho +\phi_{N})(\phi_{Y}\nu \lambda +\kappa (\phi_{L}\phi_{A}+\nu (\phi_{A}-\omega ))\right)}\right),TP_{4}=\frac{\xi \mu \rho \varrho \nu \lambda }{\phi_{X}\phi_{V}(\rho +\phi_{N})(\phi_{Y}\nu \lambda +\kappa (\phi_{L}\phi_{A}+\nu (\phi_{A}-\omega )))}.$$

Thus, if $TP_{3}>1$ and $TP_{4}>1$, then $FP_{3}$ exists. Parameters, $TP_{3}$ and $TP_{4}$ determine the situation of HTLV-1/SARS-CoV-2 dual infection. The above results can be summarized as:

Lemma 2 *There are four threshold parameters* $TP_{j}>0$ *,* j=1,2,3,4 *such that*

*(i) the infection-free fixed point,* $FP_{0}=(X^{0},0,0,0,U^{0},0,0)$ *, usually exists,*

*(ii) if* $TP_{1}>1$ *, then there is an HTLV-1 mono-infection fixed point,* $FP_{1}=(\overset{ˆ}{X},0,0,0,\overset{ˆ}{U},\overset{ˆ}{L},\overset{ˆ}{A})$ *,*

*(iii) if* $TP_{2}>1$ *, then, there is a SARS-CoV-2 mono-infection fixed point,* $FP_{2}=(\overset{˜}{X},\overset{˜}{N},\overset{˜}{Y},\overset{˜}{V},\overset{˜}{U},0,0)$ *,*

*(iv) if* $TP_{3}>1$ *and* $TP_{4}>1$ *, then, there is an HTLV-1/SARS-CoV-2 dual infection fixed point,* $FP_{3}=(\overset{¯}{X},\overset{¯}{N},\overset{¯}{Y},\overset{¯}{V},\overset{¯}{U},\overset{¯}{L},\overset{¯}{A})$ *.*

## 5 Global stability

In this part, we apply the Lyapunov approach to show the global asymptotic stability of all fixed points. Define a function G(x)≥0 as G(x)=x−1−ln⁡x. We have

(33) ln⁡x≤x−1.

According to the following finding, HTLV-1 and SARS-CoV-2 are expected to go extinct under all beginning conditions when $TP_{1}\leq 1$ and $TP_{2}\leq 1$.

Theorem 1 *If* $TP_{1}\leq 1$ *and* $TP_{2}\leq 1$ *, then* $FP_{0}=(X^{0},0,0,0,U^{0},0,0)$ *is globally asymptotically stable (GAS).*

Proof We formulate a discrete Lyapunov function $\Xi_{n}(X_{n},N_{n},Y_{n},V_{n},U_{n},L_{n},A_{n})$ as

$$\Xi_{n}=\frac{1}{B(d)}[X^{0}G\left(\frac{X_{n}}{X^{0}}\right)+N_{n}+\frac{\rho +\phi_{N}}{\rho }Y_{n}+\frac{\varrho X^{0}}{\phi_{V}}\left(1+B(d)\phi_{V}\right)V_{n}+\frac{\kappa (\rho +\phi_{N})}{\varkappa \rho }U^{0}G\left(\frac{U_{n}}{U^{0}}\right)+\frac{\kappa (\rho +\phi_{N})}{\varkappa \rho }L_{n}+\frac{\kappa (\rho +\phi_{N})(\nu +\phi_{L})}{\nu \varkappa \rho }(1+B(d)\phi_{A})A_{n}].$$

We have, $\Xi_{n}>0$ for all $X_{n}>0,N_{n}>0,Y_{n}>0,V_{n}>0,U_{n}>0,L_{n}>0,A_{n}>0$. Further, $\Xi_{n}(X^{0},0,0,0,U^{0},0,0)=0$. Computing $\Delta \Xi_{n}=\Xi_{n+1}-\Xi_{n}$ as:

$$\Delta \Xi_{n}=\frac{1}{B(d)}[X^{0}G\left(\frac{X_{n+1}}{X^{0}}\right)+N_{n+1}+\frac{\rho +\phi_{N}}{\rho }Y_{n+1}+\frac{\varrho X^{0}}{\phi_{V}}\left(1+B(d)\phi_{V}\right)V_{n+1}+\frac{\kappa (\rho +\phi_{N})}{\varkappa \rho }U^{0}G\left(\frac{U_{n+1}}{U^{0}}\right)+\frac{\kappa (\rho +\phi_{N})}{\varkappa \rho }L_{n+1}+\frac{\kappa (\rho +\phi_{N})(\nu +\phi_{L})}{\nu \varkappa \rho }(1+B(d)\phi_{A})A_{n+1}-X^{0}G\left(\frac{X_{n}}{X^{0}}\right)-N_{n}-\frac{\rho +\phi_{N}}{\rho }Y_{n}-\frac{\varrho X^{0}}{\phi_{V}}\left(1+B(d)\phi_{V}\right)V_{n}-\frac{\kappa (\rho +\phi_{N})}{\varkappa \rho }U^{0}G\left(\frac{U_{n}}{U^{0}}\right)-\frac{\kappa (\rho +\phi_{N})}{\varkappa \rho }L_{n}-\frac{\kappa (\rho +\phi_{N})(\nu +\phi_{L})}{\nu \varkappa \rho }(1+B(d)\phi_{A})A_{n}]=\frac{1}{B(d)}\left[X^{0}\left(\frac{X_{n+1}-X_{n}}{X^{0}}+\ln\left(\frac{X_{n}}{X_{n+1}}\right)\right)+\left(N_{n+1}-N_{n}\right)+\frac{\rho +\phi_{N}}{\rho }(Y_{n+1}-Y_{n}\right)+\frac{\varrho X^{0}}{\phi_{V}}\left(1+B(d)\phi_{V}\right)(V_{n+1}-V_{n})+\frac{\kappa (\rho +\phi_{N})}{\varkappa \rho }U^{0}\left(\frac{U_{n+1}-U_{n}}{U^{0}}+\ln\left(\frac{U_{n}}{U_{n+1}}\right)\right)+\frac{\kappa (\rho +\phi_{N})}{\varkappa \rho }\left(L_{n+1}-L_{n}\right)+\frac{\kappa (\rho +\phi_{N})(\nu +\phi_{L})}{\nu \varkappa \rho }(1+B(d)\phi_{A})\left(A_{n+1}-A_{n}\right)].$$

Using inequality (33) we obtain

(34) $$\Delta \Xi_{n}\leq \frac{1}{B(d)}\left[\left(X_{n+1}-X_{n}+X^{0}\left(\frac{X_{n}}{X_{n+1}}-1\right)\right)+\left(N_{n+1}-N_{n}\right)+\frac{\rho +\phi_{N}}{\rho }(Y_{n+1}-Y_{n}\right)+\frac{\varrho X^{0}}{\phi_{V}}\left(1+B(d)\phi_{V}\right)(V_{n+1}-V_{n})+\frac{\kappa (\rho +\phi_{N})}{\varkappa \rho }\left(U_{n+1}-U_{n}+U^{0}\left(\frac{U_{n}}{U_{n+1}}-1\right)\right)+\frac{\kappa (\rho +\phi_{N})}{\varkappa \rho }\left(L_{n+1}-L_{n}\right)+\frac{\kappa (\rho +\phi_{N})(\nu +\phi_{L})}{\nu \varkappa \rho }\left(1+B(d)\phi_{A}\right)\left(A_{n+1}-A_{n}\right)].$$

Inequality (34), can be written as:

$$\Delta \Xi_{n}\leq \frac{1}{B(d)}\left[\left(1-\frac{X^{0}}{X_{n+1}}\right)\left(X_{n+1}-X_{n}\right)+\left(N_{n+1}-N_{n}\right)+\frac{\rho +\phi_{N}}{\rho }(Y_{n+1}-Y_{n}\right)+\frac{\varrho X^{0}}{\phi_{V}}\left(1+B(d)\phi_{V}\right)(V_{n+1}-V_{n})+\frac{\kappa (\rho +\phi_{N})}{\varkappa \rho }\left(1-\frac{U^{0}}{U_{n+1}}\right)\left(U_{n+1}-U_{n}\right)+\frac{\kappa (\rho +\phi_{N})}{\varkappa \rho }\left(L_{n+1}-L_{n}\right)+\frac{\kappa (\rho +\phi_{N})(\nu +\phi_{L})}{\nu \varkappa \rho }\left(1+B(d)\phi_{A}\right)\left(A_{n+1}-A_{n}\right)].$$

From Eqs. (15)-(21), we have

$$\Delta \Xi_{n}\leq \left(1-\frac{X^{0}}{X_{n+1}}\right)\left(\xi -\phi_{X}X_{n+1}-\varrho V_{n}X_{n+1}\right)+\left(\varrho V_{n}X_{n+1}-(\rho +\phi_{N})N_{n+1}\right)+\frac{\rho +\phi_{N}}{\rho }(\rho N_{n+1}-\phi_{Y}Y_{n+1}-\kappa Y_{n+1}U_{n+1})+\frac{\varrho X^{0}}{\phi_{V}}(\mu Y_{n+1}-\phi_{V}V_{n+1})+\frac{\kappa (\rho +\phi_{N})}{\varkappa \rho }\left(1-\frac{U^{0}}{U_{n+1}}\right)\left(\eta +\varkappa Y_{n+1}U_{n+1}-\phi_{U}U_{n+1}-\lambda A_{n}U_{n+1}\right)+\frac{\kappa (\rho +\phi_{N})}{\varkappa \rho }\left(\lambda A_{n}U_{n+1}+\omega A_{n}-(\nu +\phi_{L})L_{n+1}\right)+\frac{\kappa (\rho +\phi_{N})(\nu +\phi_{L})}{\nu \varkappa \rho }\left(\nu L_{n+1}-\phi_{A}A_{n+1}\right)+\varrho X^{0}V_{n+1}-\varrho X^{0}V_{n}+\frac{\kappa (\rho +\phi_{N})(\nu +\phi_{L})}{\nu \varkappa \rho }\phi_{A}A_{n+1}-\frac{\kappa (\rho +\phi_{N})(\nu +\phi_{L})}{\nu \varkappa \rho }\phi_{A}A_{n}.$$

Collecting terms yields

$$\Delta \Xi_{n}\leq \left(1-\frac{X^{0}}{X_{n+1}}\right)\left(\xi -\phi_{X}X_{n+1}\right)+\left(\frac{\varrho X^{0}}{\phi_{V}}\mu -\frac{\rho +\phi_{N}}{\rho }\phi_{Y}-\frac{\rho +\phi_{N}}{\rho }\kappa U^{0}\right)Y_{n+1}+\frac{\kappa (\rho +\phi_{N})}{\varkappa \rho }\left(1-\frac{U^{0}}{U_{n+1}}\right)\left(\eta -\phi_{U}U_{n+1}\right)+\frac{\kappa (\rho +\phi_{N})}{\varkappa \rho }\left(\lambda U^{0}+\omega -\frac{\left(\nu +\phi_{L}\right)}{\nu }\phi_{A}\right)A_{n}.$$

We have

$$\xi =\phi_{X}X^{0}\;\;\text{and}\;\;\eta =\phi_{U}U^{0},$$

then we obtain

$$\Delta \Xi_{n}\leq \left(1-\frac{X^{0}}{X_{n+1}}\right)\left(\phi_{X}X^{0}-\phi_{X}X_{n+1}\right)+\frac{\kappa (\rho +\phi_{N})}{\varkappa \rho }\left(1-\frac{U^{0}}{U_{n+1}}\right)\left(\phi_{U}U^{0}-\phi_{U}U_{n+1}\right)+\left(\frac{\varrho X^{0}}{\phi_{V}}\mu -\frac{\rho +\phi_{N}}{\rho }\phi_{Y}-\frac{\rho +\phi_{N}}{\rho }\kappa U^{0}\right)Y_{n+1}+\frac{\kappa (\rho +\phi_{N})}{\varkappa \rho }\left(\lambda U^{0}+\omega -\frac{\left(\nu +\phi_{L}\right)}{\nu }\phi_{A}\right)A_{n}=-\phi_{X}\frac{{\left(X_{n+1}-X^{0}\right)}^{2}}{X_{n+1}}-\frac{\kappa (\rho +\phi_{N})}{\varkappa \rho }\phi_{U}\frac{{\left(U_{n+1}-U^{0}\right)}^{2}}{U_{n+1}}+\frac{\left(\rho +\phi_{N})(\phi_{U}\phi_{Y}+\kappa \eta \right)}{\rho \phi_{U}}\left(\frac{\varrho \xi \mu \rho \phi_{U}}{\phi_{X}\phi_{V}(\rho +\phi_{N})(\phi_{U}\phi_{Y}+\kappa \eta )}-1\right)Y_{n+1}+\frac{\kappa (\rho +\phi_{N})(\phi_{L}\phi_{A}+\nu (\phi_{A}-\omega ))}{\nu \varkappa \rho }\left(\frac{\lambda \eta \nu }{\phi_{U}(\phi_{L}\phi_{A}+\nu (\phi_{A}-\omega ))}-1\right)A_{n}=-\phi_{X}\frac{{\left(X_{n+1}-X^{0}\right)}^{2}}{X_{n+1}}-\frac{\kappa (\rho +\phi_{N})}{\varkappa \rho }\phi_{U}\frac{{\left(U_{n+1}-U^{0}\right)}^{2}}{U_{n+1}}+\frac{\left(\rho +\phi_{N})(\phi_{U}\phi_{Y}+\kappa \eta \right)}{\rho \phi_{U}}\left(TP_{2}-1\right)Y_{n+1}+\frac{\kappa (\rho +\phi_{N})(\phi_{L}\phi_{A}+\nu (\phi_{A}-\omega ))}{\nu \varkappa \rho }\left(TP_{1}-1\right)A_{n}.$$

As $TP_{1}\leq 1$ and $TP_{2}\leq 1$, $\Xi_{n}$ is monotonically decreasing. It is evident that $\Xi_{n}\geq 0$, then $\lim_{n\to \infty }\Xi_{n}\geq 0$ and thus $\lim_{n\to \infty }\Delta \Xi_{n}=0$, which provides $\lim_{n\to \infty }X_{n}=X^{0}$, $\lim_{n\to \infty }U_{n}=U^{0}$, $\lim_{n\to \infty }(TP_{2}-1)Y_{n+1}=0$ and $\lim_{n\to \infty }(TP_{1}-1)A_{n}=0$. We study four cases:

(i) $TP_{1}=1$ and $TP_{2}=1$, and then from Eq. (15),

(35) $$0=\xi -\phi_{X}X^{0}-\varrho X^{0}\lim_{n\to \infty }V_{n}\Rightarrow \lim_{n\to \infty }V_{n}=0.$$

Moreover, from Eqs. (16), (18) and (19) we get

(36) $$0=\varrho X^{0}\lim_{n\to \infty }V_{n}-(\rho +\phi_{N})\lim_{n\to \infty }N_{n+1}\Rightarrow 0=\lim_{n\to \infty }N_{n},$$

(37) $$0=\mu \lim_{n\to \infty }Y_{n+1}-\phi_{V}\lim_{n\to \infty }V_{n+1}\Rightarrow 0=\lim_{n\to \infty }Y_{n},$$

(38) $$0=\eta +\varkappa \lim_{n\to \infty }Y_{n+1}U^{0}-\phi_{U}U^{0}-\lambda \lim_{n\to \infty }A_{n}U^{0}\Rightarrow 0=\lim_{n\to \infty }A_{n}.$$

Then, from Eq. (21), we have

(39) $$0=\nu \lim_{n\to \infty }L_{n+1}-\phi_{A}\lim_{n\to \infty }A_{n+1}\Rightarrow \lim_{n\to \infty }L_{n}=0.$$

(ii) $TP_{1}=1$, $TP_{2}<1$ and $\lim_{n\to \infty }Y_{n}=0$. Eqs. (35), (36) and (38)-(39) yield $\lim_{n\to \infty }(V_{n},N_{n},A_{n},L_{n})=(0,0,0,0)$.

(iii) $TP_{1}<1$, $TP_{2}=1$ and $\lim_{n\to \infty }A_{n}=0$. Eqs. (35)-(37) and (39), give $\lim_{n\to \infty }(V_{n},N_{n},Y_{n},L_{n})=(0,0,0,0)$.

(iv) $TP_{1}<1$, $TP_{2}<1$, $\lim_{n\to \infty }Y_{n}=0$ and $\lim_{n\to \infty }A_{n}=0$. From Eqs. (35), (36) and (39) we get $\lim_{n\to \infty }(V_{n},N_{n},L_{n})=(0,0,0)$.

As a result, if $TP_{1}\leq 1$ and $TP_{2}\leq 1$, then $\lim_{n\to \infty }(X_{n},N_{n},Y_{n},V_{n},U_{n},L_{n},A_{n})=(X^{0},0,0,0,U^{0},0,0)$. This provides that, $FP_{0}$ is GAS. □

The following finding implies that, regardless of the starting circumstances, the HTLV-1 mono-infection is always established when $TP_{1}>1$ and $TP_{4}\leq 1$.

Theorem 2 *If* $TP_{1}>1$ *and* $TP_{4}\leq 1$ *then* $FP_{1}=(\overset{ˆ}{X},0,0,0,\overset{ˆ}{U},\overset{ˆ}{L},\overset{ˆ}{A})$ *is GAS.*

Proof Define

$${ϒ}_{n}=\frac{1}{B(d)}[\overset{ˆ}{X}G\left(\frac{X_{n}}{\overset{ˆ}{X}}\right)+N_{n}+\frac{\rho +\phi_{N}}{\rho }Y_{n}+\frac{\varrho \overset{ˆ}{X}}{\phi_{V}}\left(1+B(d)\phi_{V}\right)V_{n}+\frac{\kappa (\rho +\phi_{N})}{\varkappa \rho }\overset{ˆ}{U}G\left(\frac{U_{n}}{\overset{ˆ}{U}}\right)+\frac{\kappa (\rho +\phi_{N})}{\varkappa \rho }\overset{ˆ}{L}G\left(\frac{L_{n}}{\overset{ˆ}{L}}\right)+\frac{\kappa (\rho +\phi_{N})(\nu +\phi_{L})}{\nu \varkappa \rho }(1+B(d)\phi_{A})\overset{ˆ}{A}G\left(\frac{A_{n}}{\overset{ˆ}{A}}\right)].$$

Obviously ${ϒ}_{n}>0$ for all $X_{n}>0,N_{n}>0,Y_{n}>0,V_{n}>0,U_{n}>0,L_{n}>0,A_{n}>0$. Moreover, ${ϒ}_{n}(\overset{ˆ}{X},0,0,0,\overset{ˆ}{U},\overset{ˆ}{L},\overset{ˆ}{A})=0$. Computing $\Delta {ϒ}_{n}={ϒ}_{n+1}-{ϒ}_{n}$ as:

$$\Delta {ϒ}_{n}=\frac{1}{B(d)}[\overset{ˆ}{X}G\left(\frac{X_{n+1}}{\overset{ˆ}{X}}\right)+N_{n+1}+\frac{\rho +\phi_{N}}{\rho }Y_{n+1}+\frac{\varrho \overset{ˆ}{X}}{\phi_{V}}\left(1+B(d)\phi_{V}\right)V_{n+1}+\frac{\kappa (\rho +\phi_{N})}{\varkappa \rho }\overset{ˆ}{U}G\left(\frac{U_{n+1}}{\overset{ˆ}{U}}\right)+\frac{\kappa (\rho +\phi_{N})}{\varkappa \rho }\overset{ˆ}{L}G\left(\frac{L_{n+1}}{\overset{ˆ}{L}}\right)+\frac{\kappa (\rho +\phi_{N})(\nu +\phi_{L})}{\nu \varkappa \rho }(1+B(d)\phi_{A})\overset{ˆ}{A}G\left(\frac{A_{n+1}}{\overset{ˆ}{A}}\right)-\overset{ˆ}{X}G\left(\frac{X_{n}}{\overset{ˆ}{X}}\right)-N_{n}-\frac{\rho +\phi_{N}}{\rho }Y_{n}-\frac{\varrho \overset{ˆ}{X}}{\phi_{V}}\left(1+B(d)\phi_{V}\right)V_{n}-\frac{\kappa (\rho +\phi_{N})}{\varkappa \rho }\overset{ˆ}{U}G\left(\frac{U_{n}}{\overset{ˆ}{U}}\right)-\frac{\kappa (\rho +\phi_{N})}{\varkappa \rho }\overset{ˆ}{L}G\left(\frac{L_{n}}{\overset{ˆ}{L}}\right)-\frac{\kappa (\rho +\phi_{N})(\nu +\phi_{L})}{\nu \varkappa \rho }(1+B(d)\phi_{A})\overset{ˆ}{A}G\left(\frac{A_{n}}{\overset{ˆ}{A}}\right)]=\frac{1}{B(d)}[\overset{ˆ}{X}\left(\frac{X_{n+1}-X_{n}}{\overset{ˆ}{X}}+\ln\left(\frac{X_{n}}{X_{n+1}}\right)\right)+\left(N_{n+1}-N_{n}\right)+\frac{\rho +\phi_{N}}{\rho }\left(Y_{n+1}-Y_{n}\right)+\frac{\varrho \overset{ˆ}{X}}{\phi_{V}}\left(1+B(d)\phi_{V}\right)(V_{n+1}-V_{n})+\frac{\kappa (\rho +\phi_{N})}{\varkappa \rho }\overset{ˆ}{U}\left(\frac{U_{n+1}-U_{n}}{\overset{ˆ}{U}}+\ln\left(\frac{U_{n}}{U_{n+1}}\right)\right)+\frac{\kappa (\rho +\phi_{N})}{\varkappa \rho }\overset{ˆ}{L}\left(\frac{L_{n+1}-L_{n}}{\overset{ˆ}{L}}+\ln\left(\frac{L_{n}}{L_{n+1}}\right)\right)+\frac{\kappa (\rho +\phi_{N})(\nu +\phi_{L})}{\nu \varkappa \rho }(1+B(d)\phi_{A})\overset{ˆ}{A}\times \left(\frac{A_{n+1}-A_{n}}{\overset{ˆ}{A}}+\ln\left(\frac{A_{n}}{A_{n+1}}\right)\right)].$$

Using inequality (33) we get

(40) $$\Delta {ϒ}_{n}\leq \frac{1}{B(d)}[X_{n+1}-X_{n}+\overset{ˆ}{X}\left(\frac{X_{n}}{X_{n+1}}-1\right)+\left(N_{n+1}-N_{n}\right)+\frac{\rho +\phi_{N}}{\rho }\left(Y_{n+1}-Y_{n}\right)+\frac{\varrho \overset{ˆ}{X}}{\phi_{V}}\left(1+B(d)\phi_{V}\right)(V_{n+1}-V_{n})+\frac{\kappa (\rho +\phi_{N})}{\varkappa \rho }\left(U_{n+1}-U_{n}+\overset{ˆ}{U}\left(\frac{U_{n}}{U_{n+1}}-1\right)\right)+\frac{\kappa (\rho +\phi_{N})}{\varkappa \rho }\left(L_{n+1}-L_{n}+\overset{ˆ}{L}\left(\frac{L_{n}}{L_{n+1}}-1\right)\right)+\frac{\kappa (\rho +\phi_{N})(\nu +\phi_{L})}{\nu \varkappa \rho }\left(A_{n+1}-A_{n}+\overset{ˆ}{A}\left(\frac{A_{n}}{A_{n+1}}-1\right)\right)]+\frac{\kappa (\rho +\phi_{N})(\nu +\phi_{L})}{\nu \varkappa \rho }\phi_{A}A_{n+1}-\frac{\kappa (\rho +\phi_{N})(\nu +\phi_{L})}{\nu \varkappa \rho }\phi_{A}A_{n}+\frac{\kappa (\rho +\phi_{N})(\nu +\phi_{L})}{\nu \varkappa \rho }\phi_{A}\overset{ˆ}{A}\ln\left(\frac{A_{n}}{A_{n+1}}\right).$$

Inequality (40), can be written as:

$$\Delta {ϒ}_{n}\leq \frac{1}{B(d)}[\left(1-\frac{\overset{ˆ}{X}}{X_{n+1}}\right)\left(X_{n+1}-X_{n}\right)+\left(N_{n+1}-N_{n}\right)+\frac{\rho +\phi_{N}}{\rho }\left(Y_{n+1}-Y_{n}\right)+\frac{\varrho \overset{ˆ}{X}}{\phi_{V}}\left(1+B(d)\phi_{V}\right)(V_{n+1}-V_{n})+\frac{\kappa (\rho +\phi_{N})}{\varkappa \rho }\left(1-\frac{\overset{ˆ}{U}}{U_{n+1}}\right)\left(U_{n+1}-U_{n}\right)+\frac{\kappa (\rho +\phi_{N})}{\varkappa \rho }\left(1-\frac{\overset{ˆ}{L}}{L_{n+1}}\right)\left(L_{n+1}-L_{n}\right)+\frac{\kappa (\rho +\phi_{N})(\nu +\phi_{L})}{\nu \varkappa \rho }\left(1-\frac{\overset{ˆ}{A}}{A_{n+1}}\right)\left(A_{n+1}-A_{n}\right)]+\frac{\kappa (\rho +\phi_{N})(\nu +\phi_{L})}{\nu \varkappa \rho }\phi_{A}A_{n+1}-\frac{\kappa (\rho +\phi_{N})(\nu +\phi_{L})}{\nu \varkappa \rho }\phi_{A}A_{n}+\frac{\kappa (\rho +\phi_{N})(\nu +\phi_{L})}{\nu \varkappa \rho }\phi_{A}\overset{ˆ}{A}\ln\left(\frac{A_{n}}{A_{n+1}}\right).$$

From Eqs. (15)-(21) we have

$$\Delta {ϒ}_{n}\leq \left(1-\frac{\overset{ˆ}{X}}{X_{n+1}}\right)\left(\xi -\phi_{X}X_{n+1}-\varrho V_{n}X_{n+1}\right)+\left(\varrho V_{n}X_{n+1}-(\rho +\phi_{N})N_{n+1}\right)+\frac{\rho +\phi_{N}}{\rho }\left(\rho N_{n+1}-\phi_{Y}Y_{n+1}-\kappa Y_{n+1}U_{n+1}\right)+\frac{\varrho \overset{ˆ}{X}}{\phi_{V}}(\mu Y_{n+1}-\phi_{V}V_{n+1})+\varrho \overset{ˆ}{X}V_{n+1}-\varrho \overset{ˆ}{X}V_{n}+\frac{\kappa (\rho +\phi_{N})}{\varkappa \rho }\left(1-\frac{\overset{ˆ}{U}}{U_{n+1}}\right)\left(\eta +\varkappa Y_{n+1}U_{n+1}-\phi_{U}U_{n+1}-\lambda A_{n}U_{n+1}\right)+\frac{\kappa (\rho +\phi_{N})}{\varkappa \rho }\left(1-\frac{\overset{ˆ}{L}}{L_{n+1}}\right)\left(\lambda A_{n}U_{n+1}+\omega A_{n}-(\nu +\phi_{L})L_{n+1}\right)+\frac{\kappa (\rho +\phi_{N})(\nu +\phi_{L})}{\nu \varkappa \rho }\left(1-\frac{\overset{ˆ}{A}}{A_{n+1}}\right)\left(\nu L_{n+1}-\phi_{A}A_{n+1}\right)+\frac{\kappa (\rho +\phi_{N})(\nu +\phi_{L})}{\nu \varkappa \rho }\phi_{A}A_{n+1}-\frac{\kappa (\rho +\phi_{N})(\nu +\phi_{L})}{\nu \varkappa \rho }\phi_{A}A_{n}+\frac{\kappa (\rho +\phi_{N})(\nu +\phi_{L})}{\nu \varkappa \rho }\phi_{A}\overset{ˆ}{A}\ln\left(\frac{A_{n}}{A_{n+1}}\right).$$

Collecting terms we get

$$\Delta {ϒ}_{n}\leq \left(1-\frac{\overset{ˆ}{X}}{X_{n+1}}\right)\left(\xi -\phi_{X}X_{n+1}\right)-\frac{\rho +\phi_{N}}{\rho }\phi_{Y}Y_{n+1}+\frac{\varrho \overset{ˆ}{X}}{\phi_{V}}\mu Y_{n+1}+\frac{\kappa (\rho +\phi_{N})}{\varkappa \rho }\left(1-\frac{\overset{ˆ}{U}}{U_{n+1}}\right)\left(\eta -\phi_{U}U_{n+1}\right)-\frac{\kappa (\rho +\phi_{N})}{\rho }\overset{ˆ}{U}Y_{n+1}+\frac{\kappa (\rho +\phi_{N})}{\varkappa \rho }\lambda A_{n}\overset{ˆ}{U}-\frac{\kappa (\rho +\phi_{N})}{\varkappa \rho }\lambda A_{n}U_{n+1}\frac{\overset{ˆ}{L}}{L_{n+1}}+\frac{\kappa (\rho +\phi_{N})}{\varkappa \rho }\omega A_{n}-\frac{\kappa (\rho +\phi_{N})}{\varkappa \rho }\omega A_{n}\frac{\overset{ˆ}{L}}{L_{n+1}}+\frac{\kappa (\rho +\phi_{N})(\nu +\phi_{L})}{\varkappa \rho }\overset{ˆ}{L}-\frac{\kappa (\rho +\phi_{N})(\nu +\phi_{L})}{\varkappa \rho }L_{n+1}\frac{\overset{ˆ}{A}}{A_{n+1}}+\frac{\kappa (\rho +\phi_{N})(\nu +\phi_{L})}{\nu \varkappa \rho }\phi_{A}\overset{ˆ}{A}-\frac{\kappa (\rho +\phi_{N})(\nu +\phi_{L})}{\nu \varkappa \rho }\phi_{A}A_{n}+\frac{\kappa (\rho +\phi_{N})(\nu +\phi_{L})}{\nu \varkappa \rho }\phi_{A}\overset{ˆ}{A}\ln\left(\frac{A_{n}}{A_{n+1}}\right).$$

Utilizing the following conditions for $FP_{1}$:

$$\xi =\phi_{X}\overset{ˆ}{X},\;\;\;\;\;\;\eta =\phi_{U}\overset{ˆ}{U}+\lambda \overset{ˆ}{A}\overset{ˆ}{U},\lambda \overset{ˆ}{A}\overset{ˆ}{U}=(\nu +\phi_{L})\overset{ˆ}{L}-\omega \overset{ˆ}{A},\;\;\;\;\;\nu \overset{ˆ}{L}=\phi_{A}\overset{ˆ}{A},$$

we get

$$\Delta {ϒ}_{n}\leq \left(1-\frac{\overset{ˆ}{X}}{X_{n+1}}\right)\left(\phi_{X}\overset{ˆ}{X}-\phi_{X}X_{n+1}\right)+\frac{\kappa (\rho +\phi_{N})}{\varkappa \rho }\left(1-\frac{\overset{ˆ}{U}}{U_{n+1}}\right)\left(\phi_{U}\overset{ˆ}{U}-\phi_{U}U_{n+1}\right)+\left(\frac{\varrho \overset{ˆ}{X}}{\phi_{V}}\mu -\frac{\rho +\phi_{N}}{\rho }\phi_{Y}-\frac{\kappa (\rho +\phi_{N})}{\rho }\overset{ˆ}{U}\right)Y_{n+1}+\frac{\kappa (\rho +\phi_{N})}{\varkappa \rho }\lambda \overset{ˆ}{A}\overset{ˆ}{U}-\frac{\kappa (\rho +\phi_{N})}{\varkappa \rho }\lambda \overset{ˆ}{A}\overset{ˆ}{U}\frac{\overset{ˆ}{U}}{U_{n+1}}+\frac{\kappa (\rho +\phi_{N})}{\varkappa \rho }\lambda A_{n}\overset{ˆ}{U}-\frac{\kappa (\rho +\phi_{N})}{\varkappa \rho }\lambda A_{n}U_{n+1}\frac{\overset{ˆ}{L}}{L_{n+1}}+\frac{\kappa (\rho +\phi_{N})}{\varkappa \rho }\omega A_{n}-\frac{\kappa (\rho +\phi_{N})}{\varkappa \rho }\omega A_{n}\frac{\overset{ˆ}{L}}{L_{n+1}}+\frac{\kappa (\rho +\phi_{N})(\nu +\phi_{L})}{\varkappa \rho }\overset{ˆ}{L}-\frac{\kappa (\rho +\phi_{N})(\nu +\phi_{L})}{\varkappa \rho }L_{n+1}\frac{\overset{ˆ}{A}}{A_{n+1}}+\frac{\kappa (\rho +\phi_{N})(\nu +\phi_{L})}{\nu \varkappa \rho }\phi_{A}\overset{ˆ}{A}-\frac{\kappa (\rho +\phi_{N})(\nu +\phi_{L})}{\nu \varkappa \rho }\phi_{A}A_{n}+\frac{\kappa (\rho +\phi_{N})(\nu +\phi_{L})}{\nu \varkappa \rho }\phi_{A}\overset{ˆ}{A}\ln\left(\frac{A_{n}}{A_{n+1}}\right)=-\phi_{X}\frac{{\left(X_{n+1}-\overset{ˆ}{X}\right)}^{2}}{X_{n+1}}-\frac{\kappa (\rho +\phi_{N})}{\varkappa \rho }\phi_{U}\frac{{\left(U_{n+1}-\overset{ˆ}{U}\right)}^{2}}{U_{n+1}}+\left(\frac{\varrho \overset{ˆ}{X}}{\phi_{V}}\mu -\frac{\rho +\phi_{N}}{\rho }\phi_{Y}-\frac{\kappa (\rho +\phi_{N})}{\rho }\overset{ˆ}{U}\right)Y_{n+1}+\frac{\kappa (\rho +\phi_{N})}{\varkappa \rho }\lambda \overset{ˆ}{A}\overset{ˆ}{U}\left(3-\frac{\overset{ˆ}{U}}{U_{n+1}}-\frac{A_{n}U_{n+1}\overset{ˆ}{L}}{\overset{ˆ}{A}\overset{ˆ}{U}L_{n+1}}-\frac{L_{n+1}\overset{ˆ}{A}}{\overset{ˆ}{L}A_{n+1}}+\ln\left(\frac{A_{n}}{A_{n+1}}\right)\right)+\frac{\kappa (\rho +\phi_{N})}{\varkappa \rho }\omega \overset{ˆ}{A}\left(2-\frac{A_{n}\overset{ˆ}{L}}{\overset{ˆ}{A}L_{n+1}}-\frac{L_{n+1}\overset{ˆ}{A}}{\overset{ˆ}{L}A_{n+1}}+\ln\left(\frac{A_{n}}{A_{n+1}}\right)\right).$$

Using the following equalities:

$$\ln\left(\frac{A_{n}}{A_{n+1}}\right)=\ln\left(\frac{\overset{ˆ}{U}}{U_{n+1}}\right)+\ln\left(\frac{A_{n}U_{n+1}\overset{ˆ}{L}}{\overset{ˆ}{A}\overset{ˆ}{U}L_{n+1}}\right)+\ln\left(\frac{L_{n+1}\overset{ˆ}{A}}{\overset{ˆ}{L}A_{n+1}}\right),\ln\left(\frac{A_{n}}{A_{n+1}}\right)=\ln\left(\frac{A_{n}\overset{ˆ}{L}}{\overset{ˆ}{A}L_{n+1}}\right)+\ln\left(\frac{L_{n+1}\overset{ˆ}{A}}{\overset{ˆ}{L}A_{n+1}}\right).$$

We get

$$\Delta {ϒ}_{n}\leq -\phi_{X}\frac{{\left(X_{n+1}-\overset{ˆ}{X}\right)}^{2}}{X_{n+1}}-\phi_{U}\frac{\kappa (\rho +\phi_{N})}{\varkappa \rho }\frac{{\left(U_{n+1}-\overset{ˆ}{U}\right)}^{2}}{U_{n+1}}+\frac{(\rho +\phi_{N})\left[\phi_{Y}\nu \lambda +\kappa (\phi_{L}\phi_{A}+\nu (\phi_{A}-\omega ))\right]}{\nu \lambda \rho }\left(\frac{\varrho \xi \nu \lambda \rho \mu }{\phi_{X}\phi_{V}(\rho +\phi_{N})\left[\phi_{Y}\nu \lambda +\kappa (\phi_{L}\phi_{A}+\nu (\phi_{A}-\omega ))\right]}-1\right)Y_{n+1}-\frac{\kappa (\rho +\phi_{N})}{\varkappa \rho }\lambda \overset{ˆ}{A}\overset{ˆ}{U}\left[G\left(\frac{\overset{ˆ}{U}}{U_{n+1}}\right)+G\left(\frac{A_{n}U_{n+1}\overset{ˆ}{L}}{\overset{ˆ}{A}\overset{ˆ}{U}L_{n+1}}\right)+G\left(\frac{L_{n+1}\overset{ˆ}{A}}{\overset{ˆ}{L}A_{n+1}}\right)\right]-\frac{\kappa (\rho +\phi_{N})}{\varkappa \rho }\omega \overset{ˆ}{A}\left[G\left(\frac{A_{n}\overset{ˆ}{L}}{\overset{ˆ}{A}L_{n+1}}\right)+G\left(\frac{L_{n+1}\overset{ˆ}{A}}{\overset{ˆ}{L}A_{n+1}}\right)\right]=-\phi_{X}\frac{{\left(X_{n+1}-\overset{ˆ}{X}\right)}^{2}}{X_{n+1}}-\phi_{U}\frac{\kappa (\rho +\phi_{N})}{\varkappa \rho }\frac{{\left(U_{n+1}-\overset{ˆ}{U}\right)}^{2}}{U_{n+1}}+\frac{(\rho +\phi_{N})\left[\phi_{Y}\nu \lambda +\kappa (\phi_{L}\phi_{A}+\nu (\phi_{A}-\omega ))\right]}{\nu \lambda \rho }\left(TP_{4}-1\right)Y_{n+1}-\frac{\kappa (\rho +\phi_{N})}{\varkappa \rho }\lambda \overset{ˆ}{A}\overset{ˆ}{U}\left[G\left(\frac{\overset{ˆ}{U}}{U_{n+1}}\right)+G\left(\frac{A_{n}U_{n+1}\overset{ˆ}{L}}{\overset{ˆ}{A}\overset{ˆ}{U}L_{n+1}}\right)+G\left(\frac{L_{n+1}\overset{ˆ}{A}}{\overset{ˆ}{L}A_{n+1}}\right)\right]-\frac{\kappa (\rho +\phi_{N})}{\varkappa \rho }\omega \overset{ˆ}{A}\left[G\left(\frac{A_{n}\overset{ˆ}{L}}{\overset{ˆ}{A}L_{n+1}}\right)+G\left(\frac{L_{n+1}\overset{ˆ}{A}}{\overset{ˆ}{L}A_{n+1}}\right)\right].$$

As $TP_{1}>1$ and $TP_{4}\leq 1$, ${ϒ}_{n}\geq 0$ is monotonically decreasing, and there is a limit, $\lim_{n\to \infty }{ϒ}_{n}\geq 0$. Therefore, $\lim_{n\to \infty }\Delta {ϒ}_{n}=0$, which yields $\lim_{n\to \infty }X_{n}=\overset{ˆ}{X}$, $\lim_{n\to \infty }U_{n}=\overset{ˆ}{U}$, $\lim_{n\to \infty }L_{n}=\overset{ˆ}{L}$, $\lim_{n\to \infty }A_{n}=\overset{ˆ}{A}$ and $\lim_{n\to \infty }\left(TP_{4}-1\right)Y_{n}=0$. Let us address the following cases:

(i) $TP_{4}=1$. From Eq. (15), we get

(41) $$0=\xi -\phi_{X}\overset{ˆ}{X}-\varrho \overset{ˆ}{X}\lim_{n\to \infty }V_{n}\Rightarrow 0=\lim_{n\to \infty }V_{n}.$$

Moreover, from Eqs. (16) and (18) we get

(42) $$0=\varrho \overset{ˆ}{X}\lim_{n\to \infty }V_{n}-(\rho +\phi_{N})\lim_{n\to \infty }N_{n+1}\Rightarrow 0=\lim_{n\to \infty }N_{n}.$$

(43) $$0=\mu \lim_{n\to \infty }Y_{n+1}-\phi_{V}\lim_{n\to \infty }V_{n+1}\Rightarrow 0=\lim_{n\to \infty }V_{n},$$

(ii) $TP_{4}<1$ and then $\lim_{n\to \infty }Y_{n}=0$. From Eqs. (41) and (42) we obtain $\lim_{n\to \infty }N_{n}=\lim_{n\to \infty }V_{n}=0$. Hence, $FP_{1}$ is GAS. □

The following finding implies that, regardless of the starting circumstances, the SARS-CoV-2 mono-infection is confirmed as long as $TP_{2}>1$ and $TP_{3}\leq 1$.

Theorem 3 *If* $TP_{2}>1$ *and* $TP_{3}\leq 1$ *, then* $FP_{2}=(\overset{˜}{X},\overset{˜}{N},\overset{˜}{Y},\overset{˜}{V},\overset{˜}{U},0,0)$ *is GAS.*

Proof Consider

$$\Omega_{n}=\frac{1}{B(d)}[\overset{˜}{X}G\left(\frac{X_{n}}{\overset{˜}{X}}\right)+\overset{˜}{N}G\left(\frac{N_{n}}{\overset{˜}{N}}\right)+\frac{\rho +\phi_{N}}{\rho }\overset{˜}{Y}G\left(\frac{Y_{n}}{\overset{˜}{Y}}\right)+\frac{\varrho \overset{˜}{X}}{\phi_{V}}\left(1+B(d)\phi_{V}\right)\overset{˜}{V}G\left(\frac{V_{n}}{\overset{˜}{V}}\right)+\frac{\kappa (\rho +\phi_{N})}{\varkappa \rho }\overset{˜}{U}G\left(\frac{U_{n}}{\overset{˜}{U}}\right)+\frac{\kappa (\rho +\phi_{N})}{\varkappa \rho }L_{n}+\frac{\kappa (\rho +\phi_{N})(\nu +\phi_{L})}{\nu \varkappa \rho }(1+B(d)\phi_{A})A_{n}].$$

Computing $\Delta \Omega_{n}=\Omega_{n+1}-\Omega_{n}$ as:

$$\Delta \Omega_{n}=\frac{1}{B(d)}[\overset{˜}{X}G\left(\frac{X_{n+1}}{\overset{˜}{X}}\right)+\overset{˜}{N}G\left(\frac{N_{n+1}}{\overset{˜}{N}}\right)+\frac{\rho +\phi_{N}}{\rho }\overset{˜}{Y}G\left(\frac{Y_{n+1}}{\overset{˜}{Y}}\right)+\frac{\varrho \overset{˜}{X}}{\phi_{V}}\left(1+B(d)\phi_{V}\right)\overset{˜}{V}G\left(\frac{V_{n+1}}{\overset{˜}{V}}\right)+\frac{\kappa (\rho +\phi_{N})}{\varkappa \rho }\overset{˜}{U}G\left(\frac{U_{n+1}}{\overset{˜}{U}}\right)+\frac{\kappa (\rho +\phi_{N})}{\varkappa \rho }L_{n+1}+\frac{\kappa (\rho +\phi_{N})(\nu +\phi_{L})}{\nu \varkappa \rho }(1+B(d)\phi_{A})A_{n+1}-\overset{˜}{X}G\left(\frac{X_{n}}{\overset{˜}{X}}\right)-\overset{˜}{N}G\left(\frac{N_{n}}{\overset{˜}{N}}\right)-\frac{\rho +\phi_{N}}{\rho }\overset{˜}{Y}G\left(\frac{Y_{n}}{\overset{˜}{Y}}\right)-\frac{\varrho \overset{˜}{X}}{\phi_{V}}\left(1+B(d)\phi_{V}\right)\overset{˜}{V}G\left(\frac{V_{n}}{\overset{˜}{V}}\right)-\frac{\kappa (\rho +\phi_{N})}{\varkappa \rho }\overset{˜}{U}G\left(\frac{U_{n}}{\overset{˜}{U}}\right)-\frac{\kappa (\rho +\phi_{N})}{\varkappa \rho }L_{n}-\frac{\kappa (\rho +\phi_{N})(\nu +\phi_{L})}{\nu \varkappa \rho }(1+B(d)\phi_{A})A_{n}]=\frac{1}{B(d)}[\overset{˜}{X}\left(\frac{X_{n+1}-X_{n}}{\overset{˜}{X}}+\ln\left(\frac{X_{n}}{X_{n+1}}\right)\right)+\overset{˜}{N}\left(\frac{N_{n+1}-N_{n}}{\overset{˜}{N}}+\ln\left(\frac{N_{n}}{N_{n+1}}\right)\right)+\frac{\rho +\phi_{N}}{\rho }\overset{˜}{Y}\left(\frac{Y_{n+1}-Y_{n}}{\overset{˜}{Y}}+\ln\left(\frac{Y_{n}}{Y_{n+1}}\right)\right)+\frac{\varrho \overset{˜}{X}}{\phi_{V}}\left(1+B(d)\phi_{V}\right)\overset{˜}{V}\left(\frac{V_{n+1}-V_{n}}{\overset{˜}{V}}+\ln\left(\frac{V_{n}}{V_{n+1}}\right)\right)+\frac{\kappa (\rho +\phi_{N})}{\varkappa \rho }\overset{˜}{U}\left(\frac{U_{n+1}-U_{n}}{\overset{˜}{U}}+\ln\left(\frac{U_{n}}{U_{n+1}}\right)\right)+\frac{\kappa (\rho +\phi_{N})}{\varkappa \rho }\left(L_{n+1}-L_{n}\right)+\frac{\kappa (\rho +\phi_{N})(\nu +\phi_{L})}{\nu \varkappa \rho }(1+B(d)\phi_{A})\left(A_{n+1}-A_{n}\right)].$$

Using inequality (33) we obtain

(44) $$\Delta \Omega_{n}\leq \frac{1}{B(d)}[\left(X_{n+1}-X_{n}+\overset{˜}{X}\left(\frac{X_{n}}{X_{n+1}}-1\right)\right)+\left(N_{n+1}-N_{n}+\overset{˜}{N}\left(\frac{N_{n}}{N_{n+1}}-1\right)\right)+\frac{\rho +\phi_{N}}{\rho }\left(Y_{n+1}-Y_{n}+\overset{˜}{Y}\left(\frac{Y_{n}}{Y_{n+1}}-1\right)\right)+\frac{\varrho \overset{˜}{X}}{\phi_{V}}\left(V_{n+1}-V_{n}+\overset{˜}{V}\left(\frac{V_{n}}{V_{n+1}}-1\right)\right)+\frac{\kappa (\rho +\phi_{N})}{\varkappa \rho }\left(U_{n+1}-U_{n}+\overset{˜}{U}\left(\frac{U_{n}}{U_{n+1}}-1\right)\right)+\frac{\kappa (\rho +\phi_{N})}{\varkappa \rho }\left(L_{n+1}-L_{n}\right)+\frac{\kappa (\rho +\phi_{N})(\nu +\phi_{L})}{\nu \varkappa \rho }\left(A_{n+1}-A_{n}\right)]+\varrho \overset{˜}{X}V_{n+1}-\varrho \overset{˜}{X}V_{n}+\varrho \overset{˜}{X}\overset{˜}{V}\ln\left(\frac{V_{n}}{V_{n+1}}\right)+\frac{\kappa (\rho +\phi_{N})(\nu +\phi_{L})}{\nu \varkappa \rho }\phi_{A}A_{n+1}-\frac{\kappa (\rho +\phi_{N})(\nu +\phi_{L})}{\nu \varkappa \rho }\phi_{A}A_{n}.$$

Inequality (44), can be written as:

$$\Delta \Omega_{n}\leq \frac{1}{B(d)}\left[\left(1-\frac{\overset{˜}{X}}{X_{n+1}}\right)(X_{n+1}-X_{n})+\left(1-\frac{\overset{˜}{N}}{N_{n+1}}\right)(N_{n+1}-N_{n})+\frac{\rho +\phi_{N}}{\rho }\left(1-\frac{\overset{˜}{Y}}{Y_{n+1}}\right)(Y_{n+1}-Y_{n}\right)+\frac{\varrho \overset{˜}{X}}{\phi_{V}}\left(1-\frac{\overset{˜}{V}}{V_{n+1}}\right)(V_{n+1}-V_{n})+\frac{\kappa (\rho +\phi_{N})}{\varkappa \rho }\left(1-\frac{\overset{˜}{U}}{U_{n+1}}\right)(U_{n+1}-U_{n})+\frac{\kappa (\rho +\phi_{N})}{\varkappa \rho }\left(L_{n+1}-L_{n}\right)+\frac{\kappa (\rho +\phi_{N})(\nu +\phi_{L})}{\nu \varkappa \rho }\left(A_{n+1}-A_{n}\right)]+\varrho \overset{˜}{X}V_{n+1}-\varrho \overset{˜}{X}V_{n}+\varrho \overset{˜}{X}\overset{˜}{V}\ln\left(\frac{V_{n}}{V_{n+1}}\right)+\frac{\kappa (\rho +\phi_{N})(\nu +\phi_{L})}{\nu \varkappa \rho }\phi_{A}A_{n+1}-\frac{\kappa (\rho +\phi_{N})(\nu +\phi_{L})}{\nu \varkappa \rho }\phi_{A}A_{n}.$$

From Eqs. (15)-(21) we have

$$\Delta \Omega_{n}\leq \left(1-\frac{\overset{˜}{X}}{X_{n+1}}\right)(\xi -\phi_{X}X_{n+1}-\varrho V_{n}X_{n+1})+\left(1-\frac{\overset{˜}{N}}{N_{n+1}}\right)(\varrho V_{n}X_{n+1}-(\rho +\phi_{N})N_{n+1})+\frac{\rho +\phi_{N}}{\rho }\left(1-\frac{\overset{˜}{Y}}{Y_{n+1}}\right)(\rho N_{n+1}-\phi_{Y}Y_{n+1}-\kappa Y_{n+1}U_{n+1})+\frac{\varrho \overset{˜}{X}}{\phi_{V}}\left(1-\frac{\overset{˜}{V}}{V_{n+1}}\right)(\mu Y_{n+1}-\phi_{V}V_{n+1})+\frac{\kappa (\rho +\phi_{N})}{\varkappa \rho }\left(1-\frac{\overset{˜}{U}}{U_{n+1}}\right)(\eta +\varkappa Y_{n+1}U_{n+1}-\phi_{U}U_{n+1}-\lambda A_{n}U_{n+1})+\frac{\kappa (\rho +\phi_{N})}{\varkappa \rho }\left(\lambda A_{n}U_{n+1}+\omega A_{n}-(\nu +\phi_{L})L_{n+1}\right)+\frac{\kappa (\rho +\phi_{N})(\nu +\phi_{L})}{\nu \varkappa \rho }\left(\nu L_{n+1}-\phi_{A}A_{n+1}\right)+\varrho \overset{˜}{X}V_{n+1}-\varrho \overset{˜}{X}V_{n}+\varrho \overset{˜}{X}\overset{˜}{V}\ln\left(\frac{V_{n}}{V_{n+1}}\right)+\frac{\kappa (\rho +\phi_{N})(\nu +\phi_{L})}{\nu \varkappa \rho }\phi_{A}A_{n+1}-\frac{\kappa (\rho +\phi_{N})(\nu +\phi_{L})}{\nu \varkappa \rho }\phi_{A}A_{n}.$$

By collecting terms we get

$$\Delta \Omega_{n}\leq \left(1-\frac{\overset{˜}{X}}{X_{n+1}}\right)(\xi -\phi_{X}X_{n+1})-\varrho V_{n}X_{n+1}\frac{\overset{˜}{N}}{N_{n+1}}+(\rho +\phi_{N})\overset{˜}{N}-(\rho +\phi_{N})N_{n+1}\frac{\overset{˜}{Y}}{Y_{n+1}}+\frac{\rho +\phi_{N}}{\rho }\phi_{Y}\overset{˜}{Y}+\frac{\left(\rho +\phi_{N}\right)}{\rho }\kappa \overset{˜}{Y}U_{n+1}-\frac{\varrho \overset{˜}{X}}{\phi_{V}}\mu Y_{n+1}\frac{\overset{˜}{V}}{V_{n+1}}+\varrho \overset{˜}{X}\overset{˜}{V}+\varrho \overset{˜}{X}\overset{˜}{V}\ln\left(\frac{V_{n}}{V_{n+1}}\right)+\frac{\kappa (\rho +\phi_{N})}{\varkappa \rho }\left(1-\frac{\overset{˜}{U}}{U_{n+1}}\right)(\eta -\phi_{U}U_{n+1})+\left(\frac{\varrho \overset{˜}{X}}{\phi_{V}}\mu -\frac{\rho +\phi_{N}}{\rho }\phi_{Y}-\frac{\kappa (\rho +\phi_{N})}{\rho }\overset{˜}{U}\right)Y_{n+1}+\frac{\kappa (\rho +\phi_{N})}{\varkappa \rho }\left(\lambda \overset{˜}{U}+\omega -\frac{\left(\nu +\phi_{L}\right)}{\nu }\phi_{A}\right)A_{n}.$$

Using the conditions of $FP_{2}$:

$$\xi =\phi_{X}\overset{˜}{X}+\varrho \overset{˜}{V}\overset{˜}{X},\varrho \overset{˜}{V}\overset{˜}{X}=(\rho +\phi_{N})\overset{˜}{N},\rho \overset{˜}{N}=\phi_{Y}\overset{˜}{Y}+\kappa \overset{˜}{Y}\overset{˜}{U},\mu \overset{˜}{Y}=\phi_{V}\overset{˜}{V},\eta =\phi_{U}\overset{˜}{U}-\varkappa \overset{˜}{Y}\overset{˜}{U}.$$

We obtain

$$\Delta \Omega_{n}\leq \left(1-\frac{\overset{˜}{X}}{X_{n+1}}\right)(\phi_{X}\overset{˜}{X}-\phi_{X}X_{n+1})+\varrho \overset{˜}{V}\overset{˜}{X}-\varrho \overset{˜}{V}\overset{˜}{X}\frac{\overset{˜}{X}}{X_{n+1}}-\varrho \overset{˜}{V}\overset{˜}{X}\frac{V_{n}X_{n+1}\overset{˜}{N}}{\overset{ˆ}{V}\overset{˜}{X}N_{n+1}}+\varrho \overset{˜}{V}\overset{˜}{X}-\varrho \overset{˜}{V}\overset{˜}{X}\frac{N_{n+1}\overset{˜}{Y}}{\overset{˜}{N}Y_{n+1}}+\varrho \overset{˜}{V}\overset{˜}{X}-\frac{\kappa (\rho +\phi_{N})}{\rho }\overset{˜}{Y}\overset{˜}{U}+\frac{\kappa (\rho +\phi_{N})}{\rho }\overset{˜}{U}\overset{˜}{Y}\frac{U_{n+1}}{\overset{˜}{U}}-\varrho \overset{˜}{V}\overset{˜}{X}\frac{Y_{n+1}\overset{ˆ}{V}}{\overset{˜}{Y}V_{n+1}}+\varrho \overset{˜}{V}\overset{˜}{X}+\varrho \overset{˜}{V}\overset{˜}{X}\ln\left(\frac{V_{n}}{V_{n+1}}\right)+\frac{\kappa (\rho +\phi_{N})}{\varkappa \rho }\left(1-\frac{\overset{˜}{U}}{U_{n+1}}\right)(\phi_{U}\overset{˜}{U}-\phi_{U}U_{n+1})-\frac{\kappa (\rho +\phi_{N})}{\rho }\overset{˜}{Y}\overset{˜}{U}+\frac{\kappa (\rho +\phi_{N})}{\rho }\overset{˜}{Y}\overset{˜}{U}\frac{\overset{˜}{U}}{U_{n+1}}+\frac{\kappa (\rho +\phi_{N})}{\varkappa \rho }\left(\lambda \overset{˜}{U}+\omega -\frac{\left(\nu +\phi_{L}\right)}{\nu }\phi_{A}\right)A_{n}=-\phi_{X}\frac{{\left(X_{n+1}-\overset{˜}{X}\right)}^{2}}{X_{n+1}}-\phi_{U}\frac{\kappa (\rho +\phi_{N})}{\varkappa \rho }\frac{{\left(U_{n+1}-\overset{˜}{U}\right)}^{2}}{U_{n+1}}+\varrho \overset{˜}{V}\overset{˜}{X}\left(4-\frac{\overset{˜}{X}}{X_{n+1}}-\frac{V_{n}X_{n+1}\overset{˜}{N}}{\overset{˜}{V}\overset{˜}{X}N_{n+1}}-\frac{N_{n+1}\overset{˜}{Y}}{\overset{˜}{N}Y_{n+1}}-\frac{Y_{n+1}\overset{˜}{V}}{\overset{˜}{Y}V_{n+1}}+\ln\left(\frac{V_{n}}{V_{n+1}}\right)\right)-\frac{\kappa (\rho +\phi_{N})}{\rho }\overset{˜}{U}\overset{˜}{Y}\left(2-\frac{U_{n+1}}{\overset{˜}{U}}-\frac{\overset{˜}{U}}{U_{n+1}}\right)+\frac{\kappa (\rho +\phi_{N})}{\varkappa \rho }\left(\lambda \overset{˜}{U}+\omega -\frac{\left(\nu +\phi_{L}\right)}{\nu }\phi_{A}\right)A_{n}.$$

Since we have

$$-\frac{\kappa (\rho +\phi_{N})}{\rho }\overset{˜}{U}\overset{˜}{Y}\left(2-\frac{U_{n+1}}{\overset{˜}{U}}-\frac{\overset{˜}{U}}{U_{n+1}}\right)-\phi_{U}\frac{\kappa (\rho +\phi_{N})}{\varkappa \rho }\frac{{\left(U_{n+1}-\overset{˜}{U}\right)}^{2}}{U_{n+1}}=\frac{\kappa (\rho +\phi_{N})}{\rho }\overset{˜}{Y}\frac{{\left(U_{n+1}-\overset{˜}{U}\right)}^{2}}{U_{n+1}}-\phi_{U}\frac{\kappa (\rho +\phi_{N})}{\varkappa \rho }\frac{{\left(U_{n+1}-\overset{˜}{U}\right)}^{2}}{U_{n+1}}=\frac{\kappa (\rho +\phi_{N})}{\varkappa \rho }\frac{{\left(U_{n+1}-\overset{˜}{U}\right)}^{2}}{U_{n+1}}\left(\varkappa \overset{˜}{Y}-\phi_{U}\right)=-\frac{\kappa \eta (\rho +\phi_{N})}{\varkappa \rho }\frac{{\left(U_{n+1}-\overset{˜}{U}\right)}^{2}}{U_{n+1}\overset{˜}{U}}.$$

We get

(45) $$\Delta \Omega_{n}\leq -\phi_{X}\frac{{\left(X_{n+1}-\overset{˜}{X}\right)}^{2}}{X_{n+1}}-\frac{\kappa \eta (\rho +\phi_{N})}{\varkappa \rho U_{n+1}\overset{˜}{U}}{\left(U_{n+1}-\overset{˜}{U}\right)}^{2}+\varrho \overset{˜}{V}\overset{˜}{X}\left(4-\frac{\overset{˜}{X}}{X_{n+1}}-\frac{V_{n}X_{n+1}\overset{˜}{N}}{\overset{˜}{V}\overset{˜}{X}N_{n+1}}-\frac{N_{n+1}\overset{˜}{Y}}{\overset{˜}{N}Y_{n+1}}-\frac{Y_{n+1}\overset{˜}{V}}{\overset{˜}{Y}V_{n+1}}+\ln\left(\frac{V_{n}}{V_{n+1}}\right)\right)+\frac{\kappa (\rho +\phi_{N})}{\varkappa \rho }\left(\lambda \overset{˜}{U}-\frac{\phi_{L}\phi_{A}+\nu (\phi_{A}-\omega )}{\nu }\right)A_{n}.$$

By using the following equality:

$$\ln\left(\frac{V_{n}}{V_{n+1}}\right)=\ln\left(\frac{\overset{˜}{X}}{X_{n+1}}\right)+\ln\left(\frac{V_{n}X_{n+1}\overset{˜}{N}}{\overset{˜}{V}\overset{˜}{X}N_{n+1}}\right)+\ln\left(\frac{Y_{n+1}\overset{˜}{V}}{\overset{˜}{Y}V_{n+1}}\right)+\ln\left(\frac{N_{n+1}\overset{˜}{Y}}{\overset{˜}{N}Y_{n+1}}\right).$$

Then Eq. (45) becomes

$$\Delta \Omega_{n}\leq -\phi_{X}\frac{{\left(X_{n+1}-\overset{˜}{X}\right)}^{2}}{X_{n+1}}-\frac{\kappa \eta (\rho +\phi_{N})}{\varkappa \rho U_{n+1}\overset{˜}{U}}{\left(U_{n+1}-\overset{˜}{U}\right)}^{2}-\varrho \overset{˜}{V}\overset{˜}{X}\left(G\left(\frac{\overset{˜}{X}}{X_{n+1}}\right)+G\left(\frac{V_{n}X_{n+1}\overset{˜}{N}}{\overset{˜}{V}\overset{˜}{X}N_{n+1}}\right)+G\left(\frac{Y_{n+1}\overset{˜}{V}}{\overset{˜}{Y}V_{n+1}}\right)+G\left(\frac{N_{n+1}\overset{˜}{Y}}{\overset{˜}{N}Y_{n+1}}\right)\right)+\frac{\kappa (\rho +\phi_{N})}{\varkappa \rho }\left(\lambda \overset{˜}{U}-\frac{\phi_{L}\phi_{A}+\nu (\phi_{A}-\omega )}{\nu }\right)A_{n}.$$

Hence, if $TP_{3}\leq 1$, then $FP_{3}$ does not exist since $\overset{¯}{A}\leq 0$ and $\overset{¯}{L}\leq 0$. This gives

$$\frac{L_{n+1}-L_{n}}{B(d)}=\lambda A_{n}U_{n+1}+\omega A_{n}-(\nu +\phi_{L})L_{n+1}\leq 0,\frac{A_{n+1}-A_{n}}{B(d)}=\nu L_{n+1}-\phi_{A}A_{n+1}\leq 0.$$

It follows that $\left(\lambda \overset{˜}{U}-\frac{\phi_{L}\phi_{A}+\nu (\phi_{A}-\omega )}{\nu }\right)A_{n}\leq 0$ for all $A_{n}>0$, and $\lambda \overset{˜}{U}-\frac{\phi_{L}\phi_{A}+\nu (\phi_{A}-\omega )}{\nu }\leq 0$. Since, $TP_{3}\leq 1$, then $\Delta \Omega_{n}\leq 0$, for all n≥0. Therefore, $\Omega_{n}\geq 0$ is monotonically decreasing, $\lim_{n\to \infty }\Omega_{n}\geq 0$ and $\lim_{n\to \infty }\Delta \Omega_{n}=0$. It follows that, $\lim_{n\to \infty }(X_{n},N_{n},Y_{n},V_{n},U_{n})=(\overset{˜}{X},\overset{˜}{N},\overset{˜}{Y},\overset{˜}{V},\overset{˜}{U})$ and $\lim_{n\to \infty }\left(TP_{3}-1\right)A_{n}=0$. The following cases will be considered:

(i) $TP_{3}=1$ and from Eq. (21)

(46) $$0=\nu \lim_{n\to \infty }L_{n+1}-\phi_{A}\lim_{n\to \infty }A_{n+1}⟹0=\lim_{n\to \infty }L_{n}=\lim_{n\to \infty }A_{n}.$$

(ii) $TP_{3}<1$ and $\lim_{n\to \infty }A_{n}=0$. Eq. (46) imply that $\lim_{n\to \infty }L_{n}=0$. This proves that, $FP_{2}$ is GAS. □

Define a parameter *K* as:

$$K=\frac{\mu \varrho \nu \lambda \eta }{\left(\varkappa \phi_{X}\phi_{V}+\mu \varrho \phi_{U})(\phi_{L}\phi_{A}+\nu (\phi_{A}-\omega )\right)}.$$

The following finding implies that, as long as $TP_{4}>1$ and $1<TP_{3}\leq 1+K$, the HTLV-1 and SARS-CoV-2 dual infection is always found, independent of the starting circumstances.

Theorem 4 *If* $TP_{4}>1$ *and* $1<TP_{3}\leq 1+K$ *, then* $FP_{3}=(\overset{¯}{X},\overset{¯}{N},\overset{¯}{Y},\overset{¯}{V},\overset{¯}{U},\overset{¯}{L},\overset{¯}{A})$ *is GAS.*

Proof Consider

$$\Delta {Ϝ}_{n}=\frac{1}{B(d)}[\overset{¯}{X}G\left(\frac{X_{n}}{\overset{¯}{X}}\right)+\overset{¯}{N}G\left(\frac{N_{n}}{\overset{¯}{N}}\right)+\frac{\rho +\phi_{N}}{\rho }\overset{¯}{Y}G\left(\frac{Y_{n}}{\overset{¯}{Y}}\right)+\frac{\varrho \overset{¯}{X}}{\phi_{V}}\left(1+B(d)\phi_{V}\right)\overset{¯}{V}G\left(\frac{V_{n}}{\overset{¯}{V}}\right)+\frac{\kappa (\rho +\phi_{N})}{\varkappa \rho }\overset{¯}{U}G\left(\frac{U_{n}}{\overset{¯}{U}}\right)+\frac{\kappa (\rho +\phi_{N})}{\varkappa \rho }\overset{¯}{L}G\left(\frac{L_{n}}{\overset{¯}{L}}\right)+\frac{\kappa (\rho +\phi_{N})(\nu +\phi_{L})}{\nu \varkappa \rho }(1+B(d)\phi_{A})\overset{¯}{A}G\left(\frac{A_{n}}{\overset{¯}{A}}\right)].$$

Computing $\Delta {Ϝ}_{n}={Ϝ}_{n+1}-{Ϝ}_{n}$ as:

$$\Delta {Ϝ}_{n}=\frac{1}{B(d)}[\overset{¯}{X}G\left(\frac{X_{n+1}}{\overset{¯}{X}}\right)+\overset{¯}{N}G\left(\frac{N_{n+1}}{\overset{¯}{N}}\right)+\frac{\rho +\phi_{N}}{\rho }\overset{¯}{Y}G\left(\frac{Y_{n+1}}{\overset{¯}{Y}}\right)+\frac{\varrho \overset{¯}{X}}{\phi_{V}}\left(1+B(d)\phi_{V}\right)\overset{¯}{V}G\left(\frac{V_{n+1}}{\overset{¯}{V}}\right)+\frac{\kappa (\rho +\phi_{N})}{\varkappa \rho }\overset{¯}{U}G\left(\frac{U_{n+1}}{\overset{¯}{U}}\right)+\frac{\kappa (\rho +\phi_{N})}{\varkappa \rho }\overset{¯}{L}G\left(\frac{L_{n+1}}{\overset{¯}{L}}\right)+\frac{\kappa (\rho +\phi_{N})(\nu +\phi_{L})}{\nu \varkappa \rho }(1+B(d)\phi_{A})\overset{¯}{A}G\left(\frac{A_{n+1}}{\overset{¯}{A}}\right)-\overset{¯}{X}G\left(\frac{X_{n}}{\overset{¯}{X}}\right)-\overset{¯}{N}G\left(\frac{N_{n}}{\overset{¯}{N}}\right)-\frac{\rho +\phi_{N}}{\rho }\overset{¯}{Y}G\left(\frac{Y_{n}}{\overset{¯}{Y}}\right)-\frac{\varrho \overset{¯}{X}}{\phi_{V}}\left(1+B(d)\phi_{V}\right)\overset{¯}{V}G\left(\frac{V_{n}}{\overset{¯}{V}}\right)-\frac{\kappa (\rho +\phi_{N})}{\varkappa \rho }\overset{¯}{U}G\left(\frac{U_{n}}{\overset{¯}{U}}\right)-\frac{\kappa (\rho +\phi_{N})}{\varkappa \rho }\overset{¯}{L}G\left(\frac{L_{n}}{\overset{¯}{L}}\right)-\frac{\kappa (\rho +\phi_{N})(\nu +\phi_{L})}{\nu \varkappa \rho }(1+B(d)\phi_{A})\overset{¯}{A}G\left(\frac{A_{n}}{\overset{¯}{A}}\right)]=\frac{1}{B(d)}[\overset{¯}{X}\left(\frac{X_{n+1}-X_{n}}{\overset{¯}{X}}+\ln\left(\frac{X_{n}}{X_{n+1}}\right)\right)+\overset{¯}{N}\left(\frac{N_{n+1}-N_{n}}{\overset{¯}{N}}+\ln\left(\frac{N_{n}}{N_{n+1}}\right)\right)+\frac{\rho +\phi_{N}}{\rho }\overset{¯}{Y}\left(\frac{Y_{n+1}-Y_{n}}{\overset{¯}{Y}}+\ln\left(\frac{Y_{n}}{Y_{n+1}}\right)\right)+\frac{\varrho \overset{¯}{X}}{\phi_{V}}\left(1+B(d)\phi_{V}\right)\overset{¯}{V}\left(\frac{V_{n+1}-V_{n}}{\overset{¯}{V}}+\ln\left(\frac{V_{n}}{V_{n+1}}\right)\right)+\frac{\kappa (\rho +\phi_{N})}{\varkappa \rho }\overset{¯}{U}\left(\frac{U_{n+1}}{\overset{¯}{U}}-\frac{U_{n}}{\overset{¯}{U}}+\ln\left(\frac{U_{n}}{U_{n+1}}\right)\right)+\frac{\kappa (\rho +\phi_{N})}{\varkappa \rho }\overset{¯}{L}\left(\frac{L_{n+1}}{\overset{¯}{L}}-\frac{L_{n}}{\overset{¯}{L}}+\ln\left(\frac{L_{n}}{L_{n+1}}\right)\right)+\frac{\kappa (\rho +\phi_{N})(\nu +\phi_{L})}{\nu \varkappa \rho }(1+B(d)\phi_{A})\overset{¯}{A}\left(\frac{A_{n+1}}{\overset{¯}{A}}-\frac{A_{n}}{\overset{¯}{A}}+\ln\left(\frac{A_{n}}{A_{n+1}}\right)\right)].$$

Using inequality (33), we obtain

(47) $$\Delta {Ϝ}_{n}\leq \frac{1}{B(d)}[\left(X_{n+1}-X_{n}+\overset{¯}{X}\left(\frac{X_{n}}{X_{n+1}}-1\right)\right)+\left(N_{n+1}-N_{n}+\overset{¯}{N}\left(\frac{N_{n}}{N_{n+1}}-1\right)\right)+\frac{\rho +\phi_{N}}{\rho }\left(Y_{n+1}-Y_{n}+\overset{¯}{Y}\left(\frac{Y_{n}}{Y_{n+1}}-1\right)\right)+\frac{\varrho \overset{¯}{X}}{\phi_{V}}\left(V_{n+1}-V_{n}+\overset{¯}{V}\left(\frac{V_{n}}{V_{n+1}}-1\right)\right)+\frac{\kappa (\rho +\phi_{N})}{\varkappa \rho }\left(U_{n+1}-U_{n}+\overset{¯}{U}\left(\frac{U_{n}}{U_{n+1}}-1\right)\right)+\frac{\kappa (\rho +\phi_{N})}{\varkappa \rho }\left(L_{n+1}-L_{n}+\overset{¯}{L}\left(\frac{L_{n}}{L_{n+1}}-1\right)\right)+\frac{\kappa (\rho +\phi_{N})(\nu +\phi_{L})}{\nu \varkappa \rho }\left(A_{n+1}-A_{n}+\overset{¯}{A}\left(\frac{A_{n}}{A_{n+1}}-1\right)\right)]+\varrho \overset{¯}{X}V_{n+1}-\varrho \overset{¯}{X}V_{n}+\varrho \overset{¯}{X}\overset{¯}{V}\ln\left(\frac{V_{n}}{V_{n+1}}\right)+\frac{\kappa (\rho +\phi_{N})(\nu +\phi_{L})}{\nu \varkappa \rho }\phi_{A}A_{n+1}-\frac{\kappa (\rho +\phi_{N})(\nu +\phi_{L})}{\nu \varkappa \rho }\phi_{A}A_{n}+\frac{\kappa (\rho +\phi_{N})(\nu +\phi_{L})}{\nu \varkappa \rho }\phi_{A}\overset{¯}{A}\ln\left(\frac{A_{n}}{A_{n+1}}\right).$$

Inequality (47), can be written as:

$$\Delta {Ϝ}_{n}\leq \frac{1}{B(d)}\left[\left(1-\frac{\overset{¯}{X}}{X_{n+1}}\right)(X_{n+1}-X_{n})+\left(1-\frac{\overset{¯}{N}}{N_{n+1}}\right)(N_{n+1}-N_{n})+\frac{\rho +\phi_{N}}{\rho }\left(1-\frac{\overset{¯}{Y}}{Y_{n+1}}\right)(Y_{n+1}-Y_{n}\right)+\frac{\varrho \overset{¯}{X}}{\phi_{V}}\left(1-\frac{\overset{¯}{V}}{V_{n+1}}\right)(V_{n+1}-V_{n})+\frac{\kappa (\rho +\phi_{N})}{\varkappa \rho }\left(1-\frac{\overset{¯}{U}}{U_{n+1}}\right)(U_{n+1}-U_{n})+\frac{\kappa (\rho +\phi_{N})}{\varkappa \rho }\left(1-\frac{\overset{¯}{L}}{L_{n+1}}\right)(L_{n+1}-L_{n})+\frac{\kappa (\rho +\phi_{N})(\nu +\phi_{L})}{\nu \varkappa \rho }\left(1-\frac{\overset{¯}{A}}{A_{n+1}}\right)(A_{n+1}-A_{n})]+\varrho \overset{¯}{X}V_{n+1}-\varrho \overset{¯}{X}V_{n}+\varrho \overset{¯}{X}\overset{¯}{V}\ln\left(\frac{V_{n}}{V_{n+1}}\right)+\frac{\kappa (\rho +\phi_{N})(\nu +\phi_{L})}{\nu \varkappa \rho }\phi_{A}A_{n+1}-\frac{\kappa (\rho +\phi_{N})(\nu +\phi_{L})}{\nu \varkappa \rho }\phi_{A}A_{n}+\frac{\kappa (\rho +\phi_{N})(\nu +\phi_{L})}{\nu \varkappa \rho }\phi_{A}\overset{¯}{A}\ln\left(\frac{A_{n}}{A_{n+1}}\right).$$

From Eq. (15)-(21) we have

$$\Delta {Ϝ}_{n}\leq \left(1-\frac{\overset{¯}{X}}{X_{n+1}}\right)(\xi -\phi_{X}X_{n+1}-\varrho V_{n}X_{n+1})+\left(1-\frac{\overset{¯}{N}}{N_{n+1}}\right)(\varrho V_{n}X_{n+1}-(\rho +\phi_{N})N_{n+1})+\frac{\rho +\phi_{N}}{\rho }\left(1-\frac{\overset{¯}{Y}}{Y_{n+1}}\right)(\rho N_{n+1}-\phi_{Y}Y_{n+1}-\kappa Y_{n+1}U_{n+1})+\frac{\varrho \overset{¯}{X}}{\phi_{V}}\left(1-\frac{\overset{¯}{V}}{V_{n+1}}\right)(\mu Y_{n+1}-\phi_{V}V_{n+1})+\frac{\kappa (\rho +\phi_{N})}{\varkappa \rho }\left(1-\frac{\overset{¯}{U}}{U_{n+1}}\right)(\eta +\varkappa Y_{n+1}U_{n+1}-\phi_{U}U_{n+1}-\lambda A_{n}U_{n+1})+\frac{\kappa (\rho +\phi_{N})}{\varkappa \rho }\left(1-\frac{\overset{¯}{L}}{L_{n+1}}\right)(\lambda A_{n}U_{n+1}+\omega A_{n}-(\nu +\phi_{L})L_{n+1})+\frac{\kappa (\rho +\phi_{N})(\nu +\phi_{L})}{\nu \varkappa \rho }\left(1-\frac{\overset{¯}{A}}{A_{n+1}}\right)(\nu L_{n+1}-\phi_{A}A_{n+1})+\varrho \overset{¯}{X}V_{n+1}-\varrho \overset{¯}{X}V_{n}+\varrho \overset{¯}{X}\overset{¯}{V}\ln\left(\frac{V_{n}}{V_{n+1}}\right)+\frac{\kappa (\rho +\phi_{N})(\nu +\phi_{L})}{\nu \varkappa \rho }\phi_{A}A_{n+1}-\frac{\kappa (\rho +\phi_{N})(\nu +\phi_{L})}{\nu \varkappa \rho }\phi_{A}A_{n}+\frac{\kappa (\rho +\phi_{N})(\nu +\phi_{L})}{\nu \varkappa \rho }\phi_{A}\overset{¯}{A}\ln\left(\frac{A_{n}}{A_{n+1}}\right).$$

By collecting terms we get

$$\Delta {Ϝ}_{n}\leq \left(1-\frac{\overset{¯}{X}}{X_{n+1}}\right)(\xi -\phi_{X}X_{n+1})-\varrho V_{n}X_{n+1}\frac{\overset{¯}{N}}{N_{n+1}}+(\rho +\phi_{N})\overset{¯}{N}-(\rho +\phi_{N})N_{n+1}\frac{\overset{¯}{Y}}{Y_{n+1}}+\frac{\rho +\phi_{N}}{\rho }\phi_{Y}\overset{¯}{Y}+\frac{\rho +\phi_{N}}{\rho }\kappa \overset{¯}{Y}U_{n+1}-\frac{\varrho \overset{¯}{X}}{\phi_{V}}\mu Y_{n+1}\frac{\overset{¯}{V}}{V_{n+1}}+\varrho \overset{¯}{X}\overset{¯}{V}+\frac{\kappa (\rho +\phi_{N})}{\varkappa \rho }\left(1-\frac{\overset{¯}{U}}{U_{n+1}}\right)(\eta -\phi_{U}U_{n+1})+\frac{\kappa (\rho +\phi_{N})}{\varkappa \rho }\lambda A_{n}\overset{¯}{U}-\frac{\kappa (\rho +\phi_{N})}{\varkappa \rho }\lambda A_{n}U_{n+1}\frac{\overset{¯}{L}}{L_{n+1}}+\frac{\kappa (\rho +\phi_{N})}{\varkappa \rho }\omega A_{n}-\frac{\kappa (\rho +\phi_{N})}{\varkappa \rho }\omega A_{n}\frac{\overset{¯}{L}}{L_{n+1}}+\frac{\kappa (\rho +\phi_{N})}{\varkappa \rho }(\nu +\phi_{L})\overset{¯}{L}-\frac{\kappa (\rho +\phi_{N})(\nu +\phi_{L})}{\varkappa \rho }L_{n+1}\frac{\overset{¯}{A}}{A_{n+1}}+\frac{\kappa (\rho +\phi_{N})(\nu +\phi_{L})}{\nu \varkappa \rho }\phi_{A}\overset{¯}{A}+\varrho \overset{¯}{X}\overset{¯}{V}\ln\left(\frac{V_{n}}{V_{n+1}}\right)-\frac{\kappa (\rho +\phi_{N})(\nu +\phi_{L})}{\nu \varkappa \rho }\phi_{A}A_{n}+\frac{\kappa (\rho +\phi_{N})(\nu +\phi_{L})}{\nu \varkappa \rho }\phi_{A}\overset{¯}{A}\ln\left(\frac{A_{n}}{A_{n+1}}\right)+\left(\frac{\varrho \overset{¯}{X}}{\phi_{V}}\mu -\frac{\rho +\phi_{N}}{\rho }\phi_{Y}-\frac{\kappa (\rho +\phi_{N})}{\rho }\overset{¯}{U}\right)Y_{n+1}.$$

Using the conditions of $FP_{3}$:

$$\xi =\phi_{X}\overset{¯}{X}+\varrho \overset{¯}{V}\overset{¯}{X},\varrho \overset{¯}{V}\overset{¯}{X}=(\rho +\phi_{N})\overset{¯}{N},\rho \overset{¯}{N}=\phi_{Y}\overset{¯}{Y}+\kappa \overset{¯}{Y}\overset{¯}{U},\mu \overset{¯}{Y}=\phi_{V}\overset{¯}{V},\eta =\phi_{U}\overset{¯}{U}-\varkappa \overset{¯}{Y}\overset{¯}{U}+\lambda \overset{¯}{A}\overset{¯}{U},\lambda \overset{¯}{A}\overset{¯}{U}=(\nu +\phi_{L})\overset{¯}{L}-\omega \overset{¯}{A},\nu \overset{¯}{L}=\phi_{A}\overset{¯}{A},$$

we get

(48) $$\Delta {Ϝ}_{n}\leq \left(1-\frac{\overset{¯}{X}}{X_{n+1}}\right)(\phi_{X}\overset{¯}{X}-\phi_{X}X_{n+1})+\varrho \overset{¯}{V}\overset{¯}{X}-\varrho \overset{¯}{V}\overset{¯}{X}\frac{\overset{¯}{X}}{X_{n+1}}-\varrho \overset{¯}{V}\overset{¯}{X}\frac{V_{n}X_{n+1}\overset{¯}{N}}{\overset{¯}{V}\overset{¯}{X}N_{n+1}}+\varrho \overset{¯}{V}\overset{¯}{X}-\varrho \overset{¯}{V}\overset{¯}{X}\frac{N_{n+1}\overset{¯}{Y}}{\overset{¯}{N}Y_{n+1}}+\varrho \overset{¯}{V}\overset{¯}{X}-\frac{\left(\rho +\phi_{N}\right)}{\rho }\kappa \overset{¯}{Y}\overset{¯}{U}+\frac{\left(\rho +\phi_{N}\right)}{\rho }\kappa \overset{¯}{U}\overset{¯}{Y}\frac{U_{n+1}}{\overset{¯}{U}}-\varrho \overset{¯}{V}\overset{¯}{X}\frac{Y_{n+1}\overset{¯}{V}}{\overset{¯}{Y}V_{n+1}}+\varrho \overset{¯}{V}\overset{¯}{X}+\varrho \overset{¯}{X}\overset{¯}{V}\ln\left(\frac{V_{n}}{V_{n+1}}\right)+\frac{\kappa (\rho +\phi_{N})}{\varkappa \rho }\left(1-\frac{\overset{¯}{U}}{U_{n+1}}\right)(\phi_{U}\overset{¯}{U}-\phi_{U}U_{n+1})-\frac{\left(\rho +\phi_{N}\right)}{\rho }\kappa \overset{¯}{Y}\overset{¯}{U}+\frac{\left(\rho +\phi_{N}\right)}{\rho }\kappa \overset{¯}{Y}\overset{¯}{U}\frac{\overset{¯}{U}}{U_{n+1}}+\frac{\kappa (\rho +\phi_{N})}{\varkappa \rho }\lambda \overset{¯}{A}\overset{¯}{U}-\frac{\kappa (\rho +\phi_{N})}{\varkappa \rho }\lambda \overset{¯}{A}\overset{¯}{U}\frac{\overset{¯}{U}}{U_{n+1}}-\frac{\kappa (\rho +\phi_{N})}{\varkappa \rho }\lambda \overset{¯}{A}\overset{¯}{U}\frac{A_{n}U_{n+1}\overset{¯}{L}}{\overset{¯}{A}\overset{¯}{U}L_{n+1}}-\frac{\kappa (\rho +\phi_{N})}{\varkappa \rho }\omega \overset{¯}{A}\frac{A_{n}\overset{¯}{L}}{\overset{¯}{A}L_{n+1}}+\frac{\kappa (\rho +\phi_{N})}{\varkappa \rho }\lambda \overset{¯}{A}\overset{¯}{U}+\frac{\kappa (\rho +\phi_{N})}{\varkappa \rho }\omega \overset{¯}{A}-\frac{\kappa (\rho +\phi_{N})}{\varkappa \rho }\lambda \overset{¯}{A}\overset{¯}{U}\frac{L_{n+1}\overset{¯}{A}}{\overset{¯}{L}A_{n+1}}-\frac{\kappa (\rho +\phi_{N})}{\varkappa \rho }\omega \overset{¯}{A}\frac{L_{n+1}\overset{¯}{A}}{\overset{¯}{L}A_{n+1}}+\frac{\kappa (\rho +\phi_{N})}{\varkappa \rho }\lambda \overset{¯}{A}\overset{¯}{U}+\frac{\kappa (\rho +\phi_{N})}{\varkappa \rho }\omega \overset{¯}{A}+\frac{\kappa (\rho +\phi_{N})}{\varkappa \rho }\lambda \overset{¯}{A}\overset{¯}{U}\ln\left(\frac{A_{n}}{A_{n+1}}\right)+\frac{\kappa (\rho +\phi_{N})}{\varkappa \rho }\omega \overset{¯}{A}\ln\left(\frac{A_{n}}{A_{n+1}}\right)=-\phi_{X}\frac{{\left(X_{n+1}-\overset{¯}{X}\right)}^{2}}{X_{n+1}}-\frac{\kappa (\rho +\phi_{N})}{\varkappa \rho }\phi_{U}\frac{{\left(U_{n+1}-\overset{¯}{U}\right)}^{2}}{U_{n+1}}-\frac{\left(\rho +\phi_{N}\right)}{\rho }\kappa \overset{¯}{U}\overset{¯}{Y}\left(2-\frac{U_{n+1}}{\overset{¯}{U}}-\frac{\overset{¯}{U}}{U_{n+1}}\right)+\varrho \overset{¯}{V}\overset{¯}{X}\left(4-\frac{\overset{¯}{X}}{X_{n+1}}-\frac{V_{n}X_{n+1}\overset{¯}{N}}{\overset{¯}{V}\overset{¯}{X}N_{n+1}}-\frac{N_{n+1}\overset{¯}{Y}}{\overset{¯}{N}Y_{n+1}}-\frac{Y_{n+1}\overset{¯}{V}}{\overset{¯}{Y}V_{n+1}}+\ln\left(\frac{V_{n}}{V_{n+1}}\right)\right)+\frac{\kappa (\rho +\phi_{N})}{\varkappa \rho }\lambda \overset{¯}{A}\overset{¯}{U}\left(3-\frac{\overset{¯}{U}}{U_{n+1}}-\frac{A_{n}U_{n+1}\overset{¯}{L}}{\overset{¯}{A}\overset{¯}{U}L_{n+1}}-\frac{L_{n+1}\overset{¯}{A}}{\overset{¯}{L}A_{n+1}}+\ln\left(\frac{A_{n}}{A_{n+1}}\right)\right)+\frac{\kappa (\rho +\phi_{N})}{\varkappa \rho }\omega \overset{¯}{A}\left(2-\frac{A_{n}\overset{¯}{L}}{\overset{¯}{A}L_{n+1}}-\frac{L_{n+1}\overset{¯}{A}}{\overset{¯}{L}A_{n+1}}+\ln\left(\frac{A_{n}}{A_{n+1}}\right)\right).$$

Since we have

$$-\frac{\kappa (\rho +\phi_{N})}{\rho }\overset{¯}{U}\overset{¯}{Y}\left(2-\frac{U_{n+1}}{\overset{¯}{U}}-\frac{\overset{¯}{U}}{U_{n+1}}\right)-\frac{\kappa (\rho +\phi_{N})}{\varkappa \rho }\phi_{U}\frac{{\left(U_{n+1}-\overset{¯}{U}\right)}^{2}}{U_{n+1}}=\frac{\kappa (\rho +\phi_{N})}{\rho }\overset{¯}{Y}\frac{{\left(U_{n+1}-\overset{¯}{U}\right)}^{2}}{U_{n+1}}-\frac{\phi_{U}\kappa (\rho +\phi_{N})}{\varkappa \rho }\frac{{\left(U_{n+1}-\overset{¯}{U}\right)}^{2}}{U_{n+1}}=\frac{\kappa (\rho +\phi_{N})}{\rho }\frac{{\left(U_{n+1}-\overset{¯}{U}\right)}^{2}}{U_{n+1}}\left(\overset{¯}{Y}-\frac{\phi_{U}}{\varkappa }\right)=\frac{\kappa \eta (\rho +\phi_{N})\left(\varkappa \phi x\phi_{V}+\mu \varrho \phi_{U}\right)}{\mu \varrho \varkappa \rho }\frac{{\left(U_{n+1}-\overset{¯}{U}\right)}^{2}}{U_{n+1}}\left(TP_{3}-K-1\right).$$

Collecting the terms of Eq. (48), we get

(49) $$\Delta {Ϝ}_{n}\leq -\phi_{X}\frac{{\left(X_{n+1}-\overset{¯}{X}\right)}^{2}}{X_{n+1}}+\frac{\kappa \eta (\rho +\phi_{N})\left(\varkappa \phi x\phi_{V}+\mu \varrho \phi_{U}\right)}{\mu \varrho \varkappa \rho }\frac{{\left(U_{n+1}-\overset{¯}{U}\right)}^{2}}{U_{n+1}}\left(TP_{3}-K-1\right)+\varrho \overset{¯}{V}\overset{¯}{X}\left(4-\frac{\overset{¯}{X}}{X_{n+1}}-\frac{V_{n}X_{n+1}\overset{¯}{N}}{\overset{¯}{V}\overset{¯}{X}N_{n+1}}-\frac{N_{n+1}\overset{¯}{Y}}{\overset{¯}{N}Y_{n+1}}-\frac{Y_{n+1}\overset{¯}{V}}{\overset{¯}{Y}V_{n+1}}+\ln\left(\frac{V_{n}}{V_{n+1}}\right)\right)+\frac{\kappa (\rho +\phi_{N})}{\varkappa \rho }\lambda \overset{¯}{A}\overset{¯}{U}\left(3-\frac{\overset{¯}{U}}{U_{n+1}}-\frac{A_{n}U_{n+1}\overset{¯}{L}}{\overset{¯}{A}\overset{¯}{U}L_{n+1}}-\frac{L_{n+1}\overset{¯}{A}}{\overset{¯}{L}A_{n+1}}+\ln\left(\frac{A_{n}}{A_{n+1}}\right)\right)+\frac{\kappa (\rho +\phi_{N})}{\varkappa \rho }\omega \overset{¯}{A}\left(2-\frac{A_{n}\overset{¯}{L}}{\overset{¯}{A}L_{n+1}}-\frac{L_{n+1}\overset{¯}{A}}{\overset{¯}{L}A_{n+1}}+\ln\left(\frac{A_{n}}{A_{n+1}}\right)\right).$$

By using the following equality:

$$\ln\left(\frac{V_{n}}{V_{n+1}}\right)=\ln\left(\frac{\overset{¯}{X}}{X_{n+1}}\right)+\ln\left(\frac{V_{n}X_{n+1}\overset{¯}{N}}{\overset{¯}{V}\overset{¯}{X}N_{n+1}}\right)+\ln\left(\frac{N_{n+1}\overset{¯}{Y}}{\overset{¯}{N}Y_{n+1}}\right)+\ln\left(\frac{Y_{n+1}\overset{¯}{V}}{\overset{¯}{Y}V_{n+1}}\right),\ln\left(\frac{A_{n}}{A_{n+1}}\right)=\ln\left(\frac{\overset{¯}{U}}{U_{n+1}}\right)+\ln\left(\frac{A_{n}U_{n+1}\overset{¯}{L}}{\overset{¯}{A}\overset{¯}{U}L_{n+1}}\right)+\ln\left(\frac{L_{n+1}\overset{¯}{A}}{\overset{¯}{L}A_{n+1}}\right),\ln\left(\frac{A_{n}}{A_{n+1}}\right)=\ln\left(\frac{A_{n}\overset{¯}{L}}{\overset{¯}{A}L_{n+1}}\right)+\ln\left(\frac{L_{n+1}\overset{¯}{A}}{\overset{¯}{L}A_{n+1}}\right).$$

Then Eq. (49) becomes

$$\Delta {Ϝ}_{n}\leq -\phi_{X}\frac{{\left(X_{n+1}-\overset{¯}{X}\right)}^{2}}{X_{n+1}}+\frac{\kappa \eta (\rho +\phi_{N})\left(\varkappa \phi x\phi_{V}+\mu \varrho \phi_{U}\right)}{\mu \varrho \varkappa \rho }\frac{{\left(U_{n+1}-\overset{¯}{U}\right)}^{2}}{U_{n+1}}\left(TP_{3}-K-1\right)-\varrho \overset{¯}{V}\overset{¯}{X}\left[G\left(\frac{\overset{¯}{X}}{X_{n+1}}\right)+G\left(\frac{V_{n}X_{n+1}\overset{¯}{N}}{\overset{¯}{V}\overset{¯}{X}N_{n+1}}\right)+G\left(\frac{N_{n+1}\overset{¯}{Y}}{\overset{¯}{N}Y_{n+1}}\right)+G\left(\frac{Y_{n+1}\overset{¯}{V}}{\overset{¯}{Y}V_{n+1}}\right)\right]-\frac{\kappa (\rho +\phi_{N})}{\varkappa \rho }\lambda \overset{¯}{A}\overset{¯}{U}\left[G\left(\frac{\overset{¯}{U}}{U_{n+1}}\right)+G\left(\frac{A_{n}U_{n+1}\overset{¯}{L}}{\overset{¯}{A}\overset{¯}{U}L_{n+1}}\right)+G\left(\frac{L_{n+1}\overset{¯}{A}}{\overset{¯}{L}A_{n+1}}\right)\right]-\frac{\kappa (\rho +\phi_{N})}{\varkappa \rho }\omega \overset{¯}{A}\left[G\left(\frac{A_{n}\overset{¯}{L}}{\overset{¯}{A}L_{n+1}}\right)+G\left(\frac{L_{n+1}\overset{¯}{A}}{\overset{¯}{L}A_{n+1}}\right)\right].$$

We note that $\Delta {Ϝ}_{n}\leq 0$, since $1<TP_{3}\leq K+1$. Therefore, ${Ϝ}_{n}\geq 0$ is monotonically decreasing, $\lim_{n\to \infty }{Ϝ}_{n}\geq 0$ and $\lim_{n\to \infty }\Delta {Ϝ}_{n}=0$. This gives, $\lim_{n\to \infty }(X_{n},N_{n},Y_{n},V_{n},U_{n},L_{n},A_{n})=(\overset{¯}{X},\overset{¯}{N},\overset{¯}{Y},\overset{¯}{V},\overset{¯}{U},\overset{¯}{L},\overset{¯}{A})$. Hence, $FP_{3}$ is GAS. □

## 6 Numerical simulation

**Figure 1:**
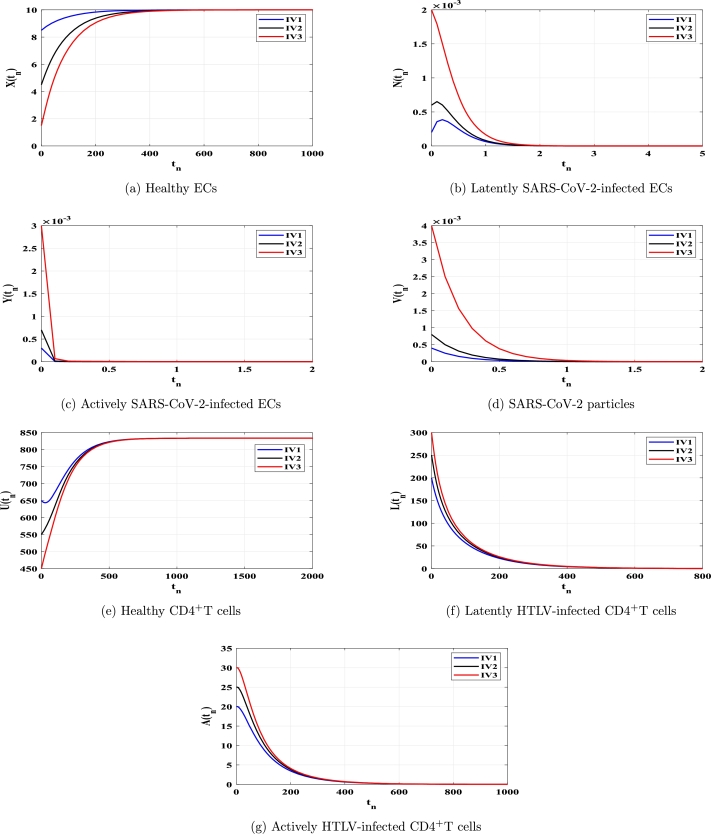
Solutions of model (15)-(21) with IV1-IV3 in case of *TP*_1_ ≤ 1 and *TP*_2_ ≤ 1.

This section uses numerical simulation to confirm the theoretical results we presented in Theorem 1, Theorem 4. We demonstrate that theoretical outcomes and numerical simulations are consistent. To conduct numerical simulation for the discrete model (15)-(21), we use the following values: ξ=0.11, $\phi_{X}=0.011$, ρ=4.08, $\phi_{N}=0.11$, $\phi_{Y}=0.11$, μ=0.24, η=10, ϰ=0.1, $\phi_{U}=0.012$, θ=0.9, $\omega^{⁎}=0.011$, ν=0.003, $\phi_{L}=0.03$, $\phi_{A}^{⁎}=0.03$ and d=0.1. The other parameters will be chosen below. We show that the solutions of system (15)-(21) with any chosen beginning circumstances (any HTLV-1/SARS-CoV-2 dual infection stage) would attack to one of the four fixed points, validating our conclusions on global stability, which are provided in Theorem 1, Theorem 4. Therefore, we take three different initial values as follows: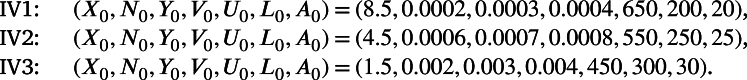 We choose *ϱ*, *κ*, $\phi_{V}$, and *λ* as:

Case (C1) ϱ=0.9, κ=1.2, $\phi_{V}=6$, and λ=0.0002. This gives $TP_{1}=0.541<1$ and $TP_{2}=0.00035<1$. Fig. 1(a-g) As seen in Fig. 1(a-g), the concentrations of healthy CD4^+^T cells and ECs rise and tend toward the healthy values $X^{0}=10$ and $U^{0}=833.33$, whereas the concentrations of other compartments fall and eventually approach zero. Consequently, $FP_{0}$ is GAS, which validates Theorem 1's conclusion. In this instance, HTLV-1 and SARS-CoV-2 are both free.

**Figure 2:**
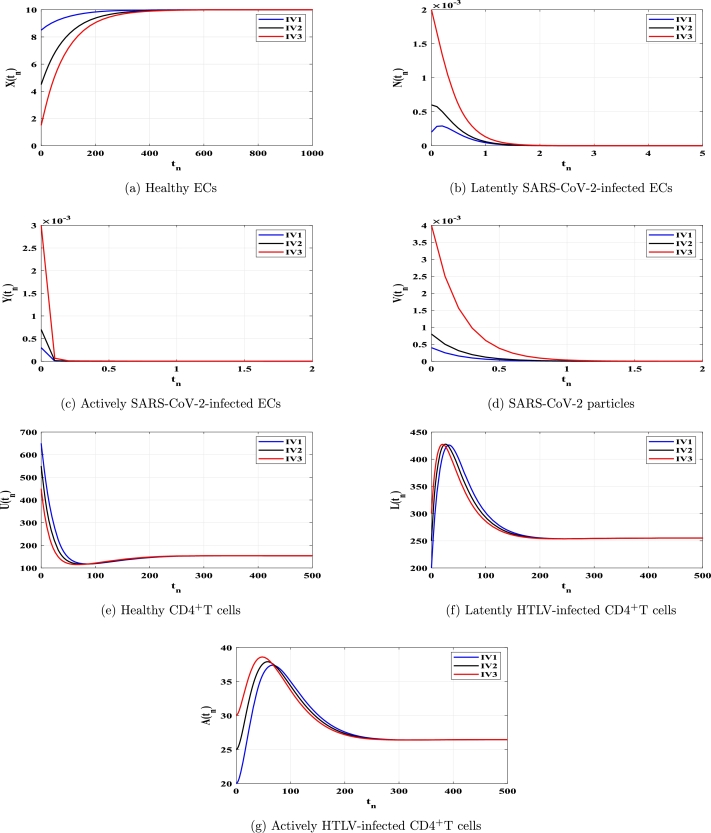
Solutions of system (15)-(21) with IV1-IV3 in case of *TP*_1_ > 1 and *TP*_4_ ≤ 1.

Case (C2) ϱ=0.6, κ=1.2, $\phi_{V}=6$, and λ=0.002. These values give $TP_{1}=5.411>1$ and $TP_{4}=0.0013<1$. Fig. 2(a-g) shows that, the system's solutions reach the fixed point $FP_{1}=(10,0,0,0,154,254.971,26.468)$. As a result, $FP_{1}$ is GAS according to Theorem 2. This presents a case of HTLV-1-infected patient who recovers from SARS-CoV-2 infection.

**Figure 3:**
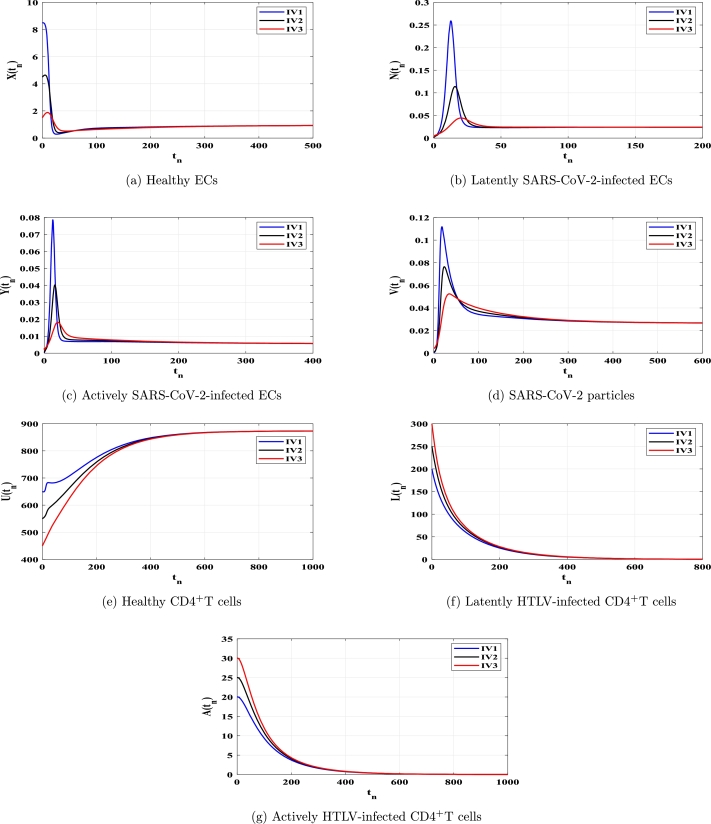
Solutions of model (15)-(21) with IV1-IV3 in case of *TP*_2_ > 1 and *TP*_3_ ≤ 1.

Case (C3) ϱ=4, κ=0.02, $\phi_{V}=0.05$, and λ=0.0002, and then $TP_{2}=11.144>1$ and $TP_{3}=0.567<1$. Fig. 3(a-g) clarifies that the system's solutions attracted to the fixed point $FP_{2}=(0.940,0.024,0.006,0.027,873.514,0,0)$ for arbitrary initial conditions. It is established by Lemma 2 and Theorem 3 that $FP_{2}$ exists and is GAS. This explains a patient's condition after being cleared of HTLV-1 infection and infected with SARS-CoV-2.

**Figure 4:**
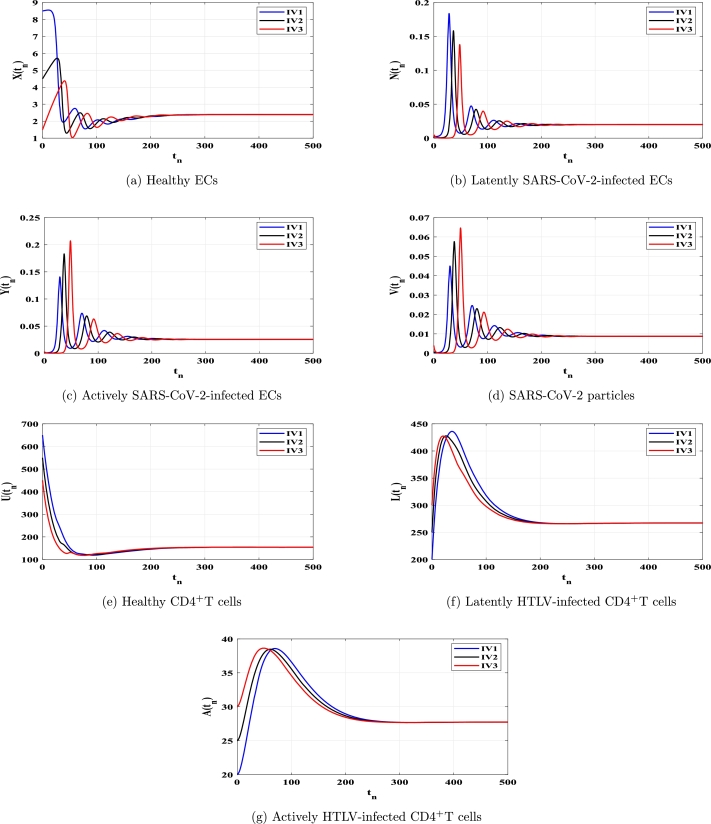
Solutions of model (15)-(21) with IV1-IV3 in case of *TP*_4_ > 1 and 1 < *TP*_3_ ≤ 1 + *K*.

Case (C4) ϱ=4, κ=0.02, $\phi_{V}=0.7$, and λ=0.002, and thus, $TP_{4}=4.186>1$, $TP_{3}=5.335>1$, and $TP_{3}<1+K=5.0722$. Fig. 4(a-g) shows that the solutions of the discrete model starting from arbitrary initials converge to the fixed point $FP_{3}=(2.389,0.020,0.026,0.009,154,267.28,27.745)$. It is established by Lemma 2 and Theorem 4 state that, $FP_{3}$ exists and is GAS.

### 6.1 Sensitivity analysis

In virology and epidemiology, sensitivity analysis is essential for simulating intricate relationships [67]. Sensitivity analysis is a useful tool for evaluating our effectiveness in stopping the virus from spreading inside and across hosts. Let us calculate the normalized forward sensitivity index of the basic reproduction numbers $TP_{1}$ and $TP_{2}$ in terms of a parameter *u* as:

(50) $$SN_{i}^{u}=\frac{u}{TP_{i}}\frac{\partial TP_{i}}{\partial u},\;\;\;\;i=1,2.$$

We utilizing the values of Case (C4), ϱ=4, κ=0.02, $\phi_{V}=0.7$, and λ=0.002.

**Figure 5:**
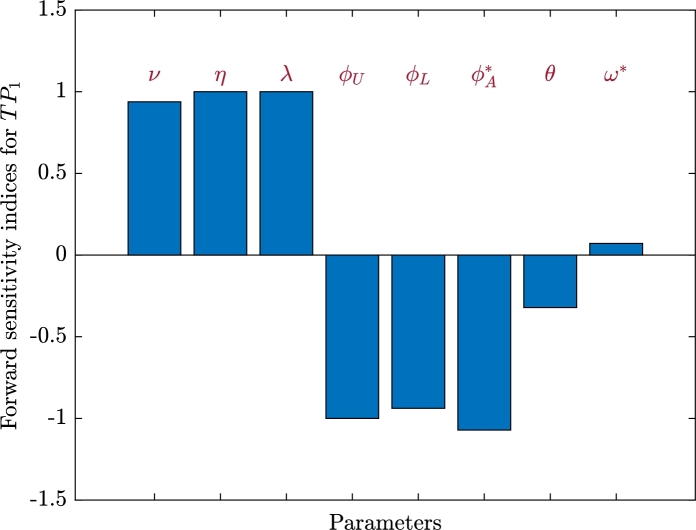
Forward sensitivity analysis of the parameters on *TP*_1_.

• Sensitivity analysis for $TP_{1}$: Table 1 and Fig. 5 display the sensitivity index $SN_{1}^{u}$. We can see that, *ν*, *η*, *λ* and $\omega^{⁎}$ have positive indices. Clearly, the most positive sensitivity indices are *η* and *λ*. When all other parameters are held constant, the advancement of HTLV-1 mono-infection in this instance is positively correlated with the parameters *ν*, *η*, *λ*, and $\omega^{⁎}$. The indices of $\phi_{U}$, $\phi_{L}$, $\phi_{A}^{⁎}$ and *θ* are all negative, indicating that as these parameters increase, $TP_{1}$ decreases. It is evident that the highest negative sensitivity indexes are $\phi_{A}^{⁎}$ and $\phi_{U}$.

**Table 1:** Sensitivity index of *TP*_1_.

*u*	$SN_{1}^{u}$	*u*	$SN_{1}^{u}$
*ν*	0.9383	*ϕ*_*L*_	−0.9383
*η*	1	$\phi_{A}^{⁎}$	−1.0714
*λ*	1	*θ*	−0.3214
*ϕ*_*U*_	−1	*ω*^⁎^	0.0714

**Figure 6:**
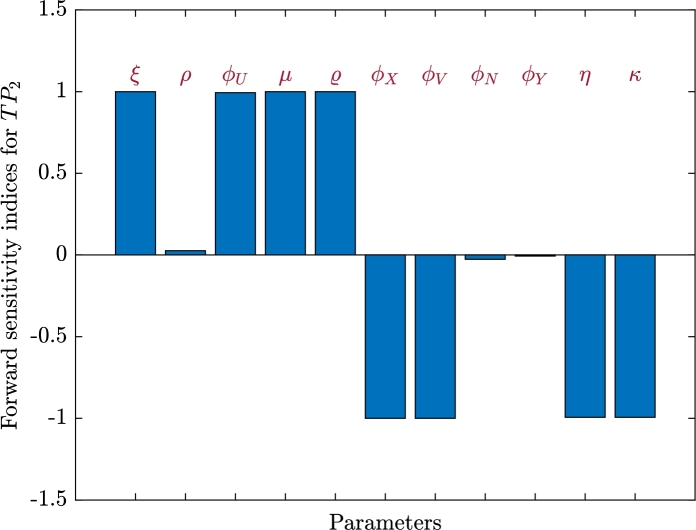
Forward sensitivity analysis of the parameters on *TP*_2_.

• Sensitivity analysis for $TP_{2}$: We display the sensitivity index, $SN_{2}^{u}$, in Table 2 and Fig. 6. We can see that, *ξ*, *ρ*, $\phi_{U}$, *μ* and *ϱ* have positive indices. It is evident that the most positive sensitivity indexes are *ξ*, *μ* and *ϱ*. The progression of SARS-CoV-2 mono-infection in this case is positively linked with the parameters *ξ*, *ρ*, $\phi_{U}$, *μ* and *ϱ* when all other parameters are maintained constant. The negative indices of $\phi_{X}$, $\phi_{V}$, $\phi_{N}$, $\phi_{Y}$, *η* and *κ* show that $TP_{2}$ falls as these parameters rise. It is clear that $\phi_{X}$ and $\phi_{V}$ have the largest negative sensitivity indices.

**Table 2:** Sensitivity index of *TP*_2_.

*u*	$SN_{2}^{u}$	*u*	$SN_{2}^{u}$	*u*	$SN_{2}^{u}$
*ξ*	1	*ϱ*	1	*ϕ*_*Y*_	−0.0066
*ρ*	0.0263	*ϕ*_*X*_	−1	*η*	−0.9934
*ϕ*_*U*_	0.9934	*ϕ*_*V*_	−1	*κ*	−0.9934
*μ*	1	*ϕ*_*N*_	−0.0263

## 7 Conclusion

In this research, a mathematical model that explains in-host dual infection with SARS-CoV-2 and HTLV-1 was built and studied. Using the NSFD approach, the nonlinear continuous-time model was discretized. We computed the model's fixed points after establishing the positivity and boundedness of the solution. The model had four fixed points, which we identified as follows: infection-free fixed point ($FP_{0}$) HTLV-1 mono-infection fixed point ($FP_{1}$), SARS-CoV-2 mono-infection fixed point ($FP_{2}$), and HTLV-1/SARS-CoV-2 dual infection fixed point ($FP_{3}$). Four threshold numbers, $TP_{j}>0$, j=1,2,3,4, were used to determine the existence and stability of the four fixed points. By building Lyapunov functions, the global stability of each fixed point in the discrete-time model was investigated. We proved that, $FP_{0}$ is GAS, when $TP_{1}\leq 1$ and $TP_{2}\leq 1$. The fixed point $FP_{1}$ exists when $TP_{1}>1$ and it is GAS when $TP_{1}>1$ and $TP_{4}\leq 1$. When $TP_{2}>1$, the fixed point $FP_{2}$ exists and it is GAS if $TP_{2}>1$ and $TP_{3}\leq 1$. Finally, we demonstrated that, the fixed point $FP_{2}$ exists and is GAS when $TP_{3}>1$ and $TP_{4}>1$. To validate the theoretical findings, we ran simulations of the discrete-time model. We demonstrated that the theoretical outcomes and numerical simulation match.

Our study's main flaw is that we were unable to use actual data to estimate the model's parameter values. It is challenging to get data for HTLV-1/SARS-CoV-2 dual-infection, even though there may be genuine data for HTLV-1 and SARS-CoV-2 mono-infections. However, the theoretical implications drawn from this work must be checked against empirical findings when actual data become available.

The model discussed in this article can be expanded by considering (i) reaction-diffusion, (ii) intracellular and immune response delays, and (iii) memory effects. Furthermore, real data will be used wherever available to provide a reliable estimate of the parameters' values. These points are left for future works.

## CRediT authorship contribution statement

**M.A. Alshaikh:** Methodology, Formal analysis, Conceptualization. **A.K. Aljahdali:** Writing – original draft, Methodology, Investigation.

## Declaration of Competing Interest

The authors declare that they have no known competing financial interests or personal relationships that could have appeared to influence the work reported in this paper.

## Data Availability

No data was used for the research described in the article.
